# Factors Affecting Access to Healthcare: An Observational Study of Children under 5 Years of Age Presenting to a Rural Gambian Primary Healthcare Centre

**DOI:** 10.1371/journal.pone.0157790

**Published:** 2016-06-23

**Authors:** Claire P. Rees, Sophie Hawkesworth, Sophie E. Moore, Bai L. Dondeh, Stefan A. Unger

**Affiliations:** 1 Centre for Primary Care & Public Health, Barts & The London School of Medicine & Dentistry, London, United Kingdom; 2 MRC International Nutrition Group, London School of Hygiene & Tropical Medicine, London, United Kingdom; 3 MRC International Nutrition Group, at MRC Keneba, MRC Unit The Gambia, Banjul, The Gambia; 4 MRC Human Nutrition Research, Elsie Widdowson Laboratory, Cambridge, United Kingdom; 5 University of Edinburgh, Department of Child Life and Health, Edinburgh, United Kingdom; Iran University of Medical Sciences, ISLAMIC REPUBLIC OF IRAN

## Abstract

**Main Objective:**

Prompt access to primary healthcare before onset of severe illness is vital to improve morbidity and mortality rates. The Gambia has high rates of child mortality and research is needed to investigate contributing factors further. This study aimed to identify factors affecting access to primary healthcare for children <5 years (y) in rural Gambia focusing on delayed presentation and severe illness at presentation as indicators in a setting where primary healthcare is delivered free of charge.

**Methods:**

Data were extracted from an electronic medical records system at a rural primary healthcare clinic in The Gambia for children (0–5y) between 2009 and 2012. First clinic attendances with malaria, lower respiratory tract infections (LRTI) and diarrhoeal disease, the main contributors to mortality in this setting, were identified and categorized as delayed/non-delayed and severe/non-severe representing our two main outcome measures. Potential explanatory variables, identified through a comprehensive literature review were obtained from an ongoing demographic surveillance system for this population. Variables associated with either delayed/non-delayed and/or with severe/non-severe presentations identified by univariate analysis (p<0.1) were assessed in multivariate models using logistic regression (p<0.05).

**Results:**

Out of 6554 clinic attendances, 571 relevant attendances were identified. Delayed presentation was common (45% of all presentations) and there was a significantly reduced risk associated with being from villages with free regular access to transport (OR 0.502, 95%CI[0.310, 0.814], p = 0.005). Children from villages with free regular transport were also less likely to present with severe illness (OR 0.557, 95%CI[0.325, 0.954], p = 0.033).

**Conclusions:**

Transport availability rather than distance to health clinic is an important barrier to accessing healthcare for children in The Gambia, and public health interventions should aim to reduce this barrier.

## Background

Barriers to accessing primary healthcare for children <5 years (y) may result in delayed presentation and severe illness with significant associated morbidity and mortality in low-income settings [1]. Reflecting on Millennium Development Goal 4 of reducing child mortality by two-thirds from 1990 to 2015 [2], significant progress has been made but unfortunately this target has not been met. More needs to be done to identify and curtail any factors preventing children receiving appropriate care. The importance of prompt presentation to healthcare facilities is highlighted in the WHO Integrated Management of Childhood Illnesses (IMCI) guidelines, which state that if families wait ‘until the child is extremely sick, or take the child to an untrained provider, the child is more likely to die from the illness’ [3]. The key role of primary healthcare to provide basic health services and public health promotion has been recognised since the Alma Ata Declaration in 1978 [4]. In The Gambia, malaria, lower respiratory infections (LRTI) and diarrhoeal disease are the major causes of child mortality in children <5y [5]. These illnesses, among others, require prompt treatment to prevent clinical deterioration [6–8] and thus identification of barriers to healthcare access is paramount.

A literature review of known factors affecting access to healthcare for children in Sub-Saharan Africa (SSA) identified 38 original articles and one systematic review (see S2 File (PRISMA flow diagram), Tables 1 and 2). Previous studies provided conflicting findings and had several limitations (see S4 File, Table 2), which this study in Kiang West (KW), The Gambia aims to address. Most studies focused on outcomes of healthcare utilization[9–24] and child mortality[25–40], rather than on delayed presentation and illness severity with the exception of malaria[41–46]. Studies focusing on healthcare utilization described illness episodes without presentation to a healthcare facility and relied on parental recall of previous illness over 7 days to 1 month prior to survey. In contrast, our study focused on delayed/non-delayed presentation and severe/non-severe illness at presentation as indicators of access to healthcare rather than utilization *per se*.

**Table 1:** PICO used for literature search.

**Population**	Children <5y in Sub-Saharan Africa
**Exposure Groups and Comparison**	Distance to health facility, SE factors, maternal education, age of child, age of mother, gender, transport cost, transport availability, transport time, number of siblings, death of mother, death of sibling, seasonality
**Outcome**	Child mortality or Decreased healthcare utilization or Delayed presentation or Severe illness at presentation

**Table 2:** Potential explanatory variables and evidence from literature review.

Variable	Evidence of association	Inconclusive or no evidence
**Child’s gender**	Higher mortality for males[29]; more likely to have travelled further to seek care if male[22]; lower hospitalisation rates for females, males experienced reduced distance decay effect [31].	No difference in mortality[27, 32, 38]; gender did not affect healthcare utilization; gender not associated with malaria severity[46]; gender not associated with prompt treatment[41].
**Child’s age**	Younger children travelled further than older children to reach clinic[10]; longer delays for children seeking malaria treatment in older children[41]; older children had lower mortality[29, 35]; less likely to seek care for older children[17].	No difference in mortality[32]; mixed effects of child’s age on utilization[15]; age of child not related to prompt treatment[45]; severity of malaria not related to age[46].
**Mother’s age**	Increased mortality with mother’s <20y[25]; infant mortality higher for teenage mothers[27]; increased child mortality if mother <18y[26]; higher healthcare utilization by younger mothers[15]; increased mortality with younger mother[37]; mothers >35y less likely to take children to seek care[17].	Malaria severity not associated with mother’s age[46].
**Death of maternal sibling**	Death of sibling associated with increased mortality[27, 26, 29]; increased mortality risk if 2+ dead siblings[28]; more likely to be delayed if no history of sibling death[42].	No association with child mortality[37].
**Death of mother**	Death of child’s mother associated with increased mortality[27, 26, 29, 39].	N/I
**Transport cost**	Higher transport cost resulted in delayed malaria treatment[42]; small effect of transport cost on healthcare utilization[23].	No impact of transport cost on child mortality[33].
**Mother attended English school**	Higher infant mortality if the mother had no formal education [27]; increased utilization with higher maternal education[10, 35, 43]; more likely to receive prompt antimalarial treatment if had maternal education[44]; increased mortality if lower maternal education[18].	No association with child mortality[37, 32]; maternal education did not affect utilization[19, 45]; maternal education not associated with prompt malaria treatment[41].
**Parents are monogamous (mother has no co-wives)**	Delayed treatment of malaria was associated with parents being in a monogamous marriage[42].	N/I
**Severe illness at presentation**	More likely to attend health facility if symptoms of severe illness[11]; children with severe illness travelled further than those with non-severe illness[10]; children with severe pneumonia more likely to be taken to health facility[15]; prompt presentation associated with signs of severe malaria[41]; more likely to seek care for higher fevers[20]; children with diarrhoea who had lethargy more likely to be taken to health centre[12]; carer perceived illness severity influence likelihood of seeking care, more likely to seek care for diarrhoea than cough[36].	Severity of illness did not influence utilization[45].
**Symptom duration**	Children who died in hospital with diarrhoea presented with longer duration of symptoms[32]; decreased utilization if fever <5 days[35]; children who died from malaria had longer symptom duration before presentation[46].	No association with fever duration and visiting a health centre[20].
**Distance to clinic (calculated for route most commonly used)**	Delayed presentation with malaria associated with living >3km from health facility[42]; increased utilization if closer to healthcare facility, increased infant mortality if >5km from facility[27]; increased infant mortality if living >10km away[26]; increased utilization with decreased distance[23, 10, 18]; steady decline in utilization up to 6km[16]; increased mortality if >5km away[29, 30]; more likely to seek care if clinic in village of residence or hospital nearby[24].	No association with prompt treatment in malaria[45]; distance to health facility <5km vs. >5km no significant effect on child mortality[28]; healthcare utilization not associated with distance to clinic[21]; distance did not affect child mortality[33, 35]; distance not associated with severe presentation of malaria[46].
**Travel time**	Increased mortality with increasing walking time to clinic[36]; utilization decreased steadily from 3hrs travel time onwards[14]; increased utilization with decreased travel time[23]; travel time >3hrs associated with decreased utilization and increased mortality[22]; hospitalisation rates decreased and mortality rates increased with increasing travel time[31]; increased rates of hospitalisation with increased walking time to primary care clinic[48].	No impact of travel time on child mortality[33]; travel time not associated with severe presentation of malaria[46].
**Only child (no maternal siblings)**	Increased mortality risk being an only child or caregiver not looking after any other children[33]; increased chance of prompt treatment of malaria if only one child <5y[44].	N/I
**Number of maternal siblings**	Maternal parity >5 has been linked to increased child mortality[28]; increased mortality if 4+ children <5y in household[39]; decreased utilization with increasing number of children <6y in household[41].	N/I
**Birth order**	Mortality increased for first-born infants[27, 26].	N/I

N/I- no evidence identified

Data on clinic presentation were extracted from an electronic medical records system rather than often unreliable paper documentation used in previous studies and seen in similar settings[47]. Clinical data was linked with a Demographic Surveillance System (DSS) maximizing data accuracy and allowing investigation of family factors for which limited evidence in relation to access to health exist (Table 2) such as number of siblings, history of death of a sibling or mother, child and maternal age and maternal education. We were able to measure distance to clinic using GPS accurate measurements along routes most commonly taken and assess distance as a continuous variable, whereas most previous studies measured straight-line (Euclidean) distance or categorized distance as a binary variable[27, 28, 10, 42, 16, 29, 30, 12, 33, 35, 45, 38, 46]. Only five other studies were identified using distance/travel time as a continuous variable[36, 31, 41, 14, 48].

## Methods

### Study setting

**Fig 1:**
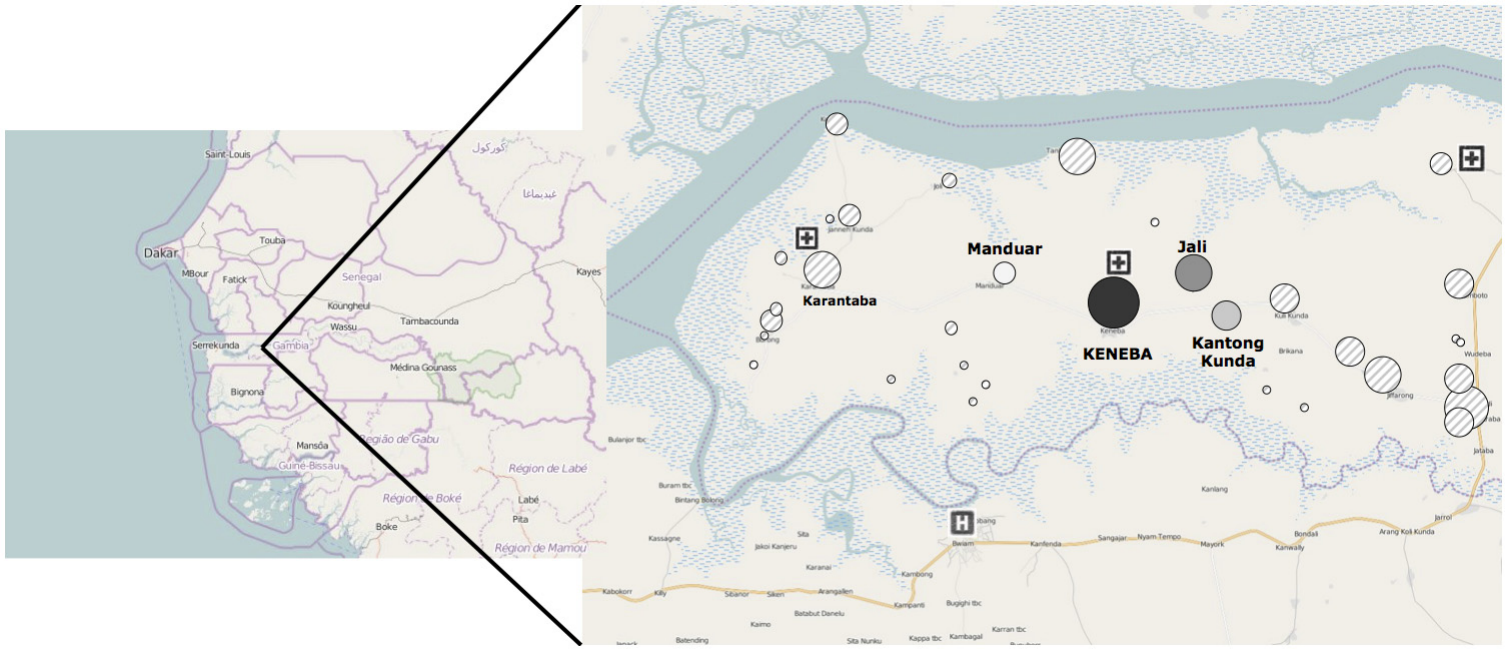
Study setting: Kiang West in the Lower River Division district, The Gambia, West Africa. Circles represent the size of population comparatively. The primary healthcare clinic is situated in Keneba. ‘Core villages’ provided with free transport include Keneba, Manduar and Kantong Kunda (black circles). Two small government run health posts in KW and the nearest hospital in Bwiam out with KW are indicated.

Keneba is a rural village in KW, an isolated district of the Lower River Region, The Gambia (Fig 1). Mandinka is the major ethnic group (Mandinka 79.9%, Fula 16.2%, Jola 2.4%, other 1.3%). Literacy rates among women aged 15–49 in The Gambia are 45.0% (ranging from 64.8% for those aged 15–19 to 15.7% for those aged 45–49[49]. Most people are subsistence farmers, and both men and women are engaged in agricultural activities. The population is predominantly Muslim with polygamous marriages common. People live in houses grouped into compounds, few have access to electricity and there were no paved roads at the time of this study. Free healthcare provision at Keneba in KW (run by the Medical Research Council (MRC) The Gambia unit) has existed since 1975 and hence this setting allows an analysis of what barriers remain when healthcare is provided free of charge[50]. There has been no major research undertaken into barriers of access to healthcare in the described cohort. Limited studies exist in settings with free healthcare provision and hence this study was conducted[27, 48]. At the MRC-run clinic in Keneba, all children <5y are triaged by a nurse and then reviewed by a doctor[50]. The nurses received formal training in the use of the triage system with regular updates and weekly clinical meetings. It is possible to observe the child for up to 24 hours; if further inpatient care is needed they are referred to a hospital outside KW. There are two government-run clinics in KW (Karantaba and Kwinella), however there are no doctors at these clinics and supplies of medication are unreliable (Fig 1)[50].

The population of the three rural villages of Keneba, Manduar and Kantong Kunda (overall population of around 3000 with 16% under 5y) (Fig 1), known as the ‘core villages’, has been documented in longitudinal demographic and health surveys for more than 50 years [51]. An MRC vehicle provides free transportation to the clinic in Keneba from Manduar and Kantong Kunda Monday to Friday. Also, weekly child welfare clinics that provide childhood vaccinations are run for children under 3 years from these ‘core villages’[50].

### Study population

The study cohort included children under 5y resident in KW who self-referred to the Keneba clinic between November 2009 and April 2012. Children within this cohort were initially recruited for an unrelated study (See S1 File for details of the recruitment process). Children were excluded from this study if a) they lived outside KW or were not registered with the Kiang West Demographic Surveillance System (KWDSS) during the study period as we would not be able to correlate demographic data for them, and b) presented as part of child welfare, follow up or research clinics as these were not initial presentations representing a decision by the carergiver. Ethical approval for the study was granted by the London School of Hygiene and Tropical Medicine Ethics Board and the joint Gambia Government / MRC Unit The Gambia Ethics Committee (L2011.33). Informed oral and written consent was obtained from the guardian of each participating child to use data collected confidentially and identifiable by an identification number only. If guardians were illiterate the consent form would be read out by a trained field assistant and a thumbprint used to indicate full understanding and agreement once understanding had been checked. MRC Keneba has an agreement with the study population in Kiang West (KW) to undertake research studies and, prior to implementation, all studies must seek full approved by the joint Gambia Government/MRC Unit The Gambia Ethics Committee. At the community level, regular meetings between the MRC and local community are held in particular before any major study to get their support. In return the KW population receive free health care at MRC Keneba health clinic. There is an excellent relationship between MRC and the KW population, as recently described by Hennig, Unger at al.[50].

### Data extraction and cleaning

Each resident in KW has a unique identification number, the West Kiang Number (WKNo) providing the linkage between KWDSS and other database platforms including the Keneba Electronic Medical Records System (KEMReS). Since 2009 KEMReS captured data on all clinic presentations to the Keneba clinic, including presenting complaint, physical examination findings, vital signs, laboratory investigations, diagnoses and treatment [52, 50]. Full details on coverage, completeness and data quality attributes of KEMReS and the KWDSS can be found elsewhere (http://ing.mrc.ac.uk/home/research-areas/keneba-electronic-medical-records-system-kemres/ and [50].

Using only the WKNo for identification of the children included, data on all their self-referred clinic presentations between 11 November 2009 and 02 April 2012 were extracted from KEMReS and compiled onto an Excel Microsoft spreadsheet. Clinic visits meeting the WHO criteria for a diagnosis of malaria, LRTI or diarrhoeal disease were then identified [53] (Table 3) The KEMReS database included a selected list ICD-10 codes based on disease occurrence and availability of laboratory confirmatory tests available. (see S15 Table for a list of ICD-10 codes). We included the first attendances with each disease in each individual dataset even if they were co-diagnosis entries. Repeat attendances were excluded. Due to small numbers for each individual disease entity, focus was directed at combined attendances with either malaria, LRTI or diarrhoea. Where a child attended more than once with a different disease only their first attendance was counted in the analysis of combined first attendances. In co-diagnosis attendances, the illness severity and symptom duration was chosen from the malaria, then LRTI, then diarrhoeal disease dataset in order of preference. Paper records were referred to if there were any missing data. It was then determined if each case was a delayed or prompt presentation and whether the presentation was with severe illness (see Table 4 for definitions).

**Table 3:** Inclusion, exclusion and diagnosis selection criteria for each dataset *.

	Inclusion criteria (ICD-10 codes in brackets[54])	Definition- if not met, entry excluded
**Malaria dataset**	Diagnosis of ‘malaria (free text)’ or ‘plasmodium falciparum malaria (B50)’ or positive blood film	History of fever or fever on presentation AND positive blood film
**LRTI dataset**	Diagnosis of ‘pneumonia (J18)’ or ‘bronchiolitis (J21)’ or ‘Acute bronchitis (J20)’ or ‘acute respiratory infection (free text)’ or ‘resolving pneumonia (free text)’	Cough or shortness of breath or chest pain or breathless AND raised RR or crackles or crepitations or decreased air entry or wheeze or signs of severe disease i.e. chest indrawing or nasal flaring or grunting or signs of dehydration or head nodding or lethargy or decreased coma score
**Diarrhoeal disease dataset**	Diagnoses- ‘unspecified bacterial intestinal infection (A04.9)’ or ‘giardia (A07.1)’ or ‘viral gastroenteritis (A08)’ or ‘diarrhea (free text)’ or ‘diarrheal disease (free text)’ or ‘non infective diarrhoea (K52.9)’	Diarrhoea listed as a presenting complaint

*Numbers in brackets are ICD-10 codes

**Table 4:** Severity score criteria.

	Criteria				
	History and examination (One of the following)	Clinician impression	Parenteral treatment	Outcome	Early warning score
**Malaria**	Lethargy, convulsions, behaviour change, weak, not standing, apathetic, decreased coma score, jaundice, pallor, Hb <5g/dl, haematocrit <15%, blood glucose <2.5 mmol/L	Acutely ill looking	IV/IM quinine	Observation or referral	3 or more points
**LRTI**	Cyanosis, convulsions, lethargy, decreased coma score, behavioural change, head nodding, signs of dehydration, chest wall indrawing, nasal flaring, grunting	Acutely ill looking	IV antibiotics	Observation or referral	3 or more points
**Diarrhoeal disease**	Weak, not standing, lethargy, decreased coma score, behavioural change, not drinking/breastfeeding, signs of dehydration	Acutely ill looking	IV fluids	Observation or eferral	3 or more points

### Definition of delayed presentation

Symptom duration was reported by the caregiver and recorded in KEMReS. The start of illness was defined as onset of fever for malaria, onset of diarrhoea for diarrhoeal disease and onset of cough or shortness of breath, whichever came first, for LRTI. Delayed presentation was defined as symptom duration of 3 days or more.

### Definition of severe illness

Illness severity was calculated using a number of criteria. For each of these that were fulfilled one point was awarded giving an overall severity score. A cut-off score of 2 or above out of 5 was chosen to indicate severe illness to increase the specificity (Table 4). The criteria included 1) history and examination findings meeting WHO criteria for severe illness, 2) if the clinician felt they were acutely ill looking, 3) if they required parenteral treatment, 4) if they required observation or referral, and 5) if they had a positive early warning score of 3+ out of 5. The criteria included an early warning score, as it is known that triage observations offer a valuable insight into the severity of illness [55, 56]. This study used the Paediatric Advanced Warning Score (PAWS), developed in 2008 by Egdell et al [57] together with Advanced Paediatric Life Support guidelines [58]. This PAWS scoring system has been validated in an accident and emergency setting in the UK, with a sensitivity of 70% and specificity of 90% for children needing intensive care admission [57]. This system was chosen as the observations needed to calculate the score were available from KEMReS or easily modified. Points were given for abnormal observations and a score of 3 or greater indicates severe illness (see S1 Table).

One point was given for each entity. The WHO definitions [53] of severe malaria, severe pneumonia and bronchiolitis and severe diarrhoeal illness were adapted using equivalent criteria documented in the KEMReS database. Clinicians documented if the child was ‘acutely ill-looking’. Although subjective, this provides a useful overview of how the clinician felt the patient’s condition was. The use of parenteral treatment suggests severe disease, as it is the recommended treatment for severe illness [53]. In severe cases a child would be observed in the clinic observation bay or referred to a hospital. The early warning score was based on observations at triage and was adapted to the setting.

### Explanatory variables

A list of potential explanatory variables was compiled from identified factors in an extensive literature review and through discussion between the researchers based on clinical experience (see Tables 1 and 2 and S2–S4 Files). Available data on potential explanatory variables that could present a barrier to accessing healthcare were extracted in February 2012 from KWDSS, which covers the entire KW district. The KWDSS data was used from two collection dates, for attendances 11 Nov 09–18 Jun 11 data was used from the June 2011 survey and for attendances 19 Jul 11–02 Apr 12 data was used from the March 2012 survey.

### Data analysis

The datasets were imported into Stata 11.0 for statistical analysis. Where data on potential explanatory variables were missing they were excluded from analysis of that variable. Univariate analyses were conducted to assess the association of potential explanatory variables with delayed presentation and to assess the association with severe illness for a dataset of combined first attendances (see analyses of individual disease datasets in S2–S10 Tables). Numerical continuous explanatory variables, ordered categorical variables and dichotomous explanatory variables were analysed using the t-test, the Kruskal-Wallis oneway analysis, and the Chi-squared test (or Fisher’s exact test for small sample sizes) respectively. Where statistically significant associations were observed in univariate analyses at the p<0.1 level, they were entered into multivariate logistic regression models to adjust for potential confounding. The alpha level chosen for statistical significance was 5%. All logistic regression analyses were also adjusted for seasonality and year of attendance. Graphing, including lowess curves and tables were used to verify the binary logistic regression models. Variables identified in the univariate analysis (p<0.1) were entered sequentially into the models. Models were compared using graph of observed versus predicted p and the goodness of fit was assessed using Hosmer-Lemeshow goodness-of-fit test. In order to assess for bias, we ran a sensitivity analysis including clinical attendances with no diagnosis but which may have had one of the three target disease studied.

## Results

**Fig 2:**
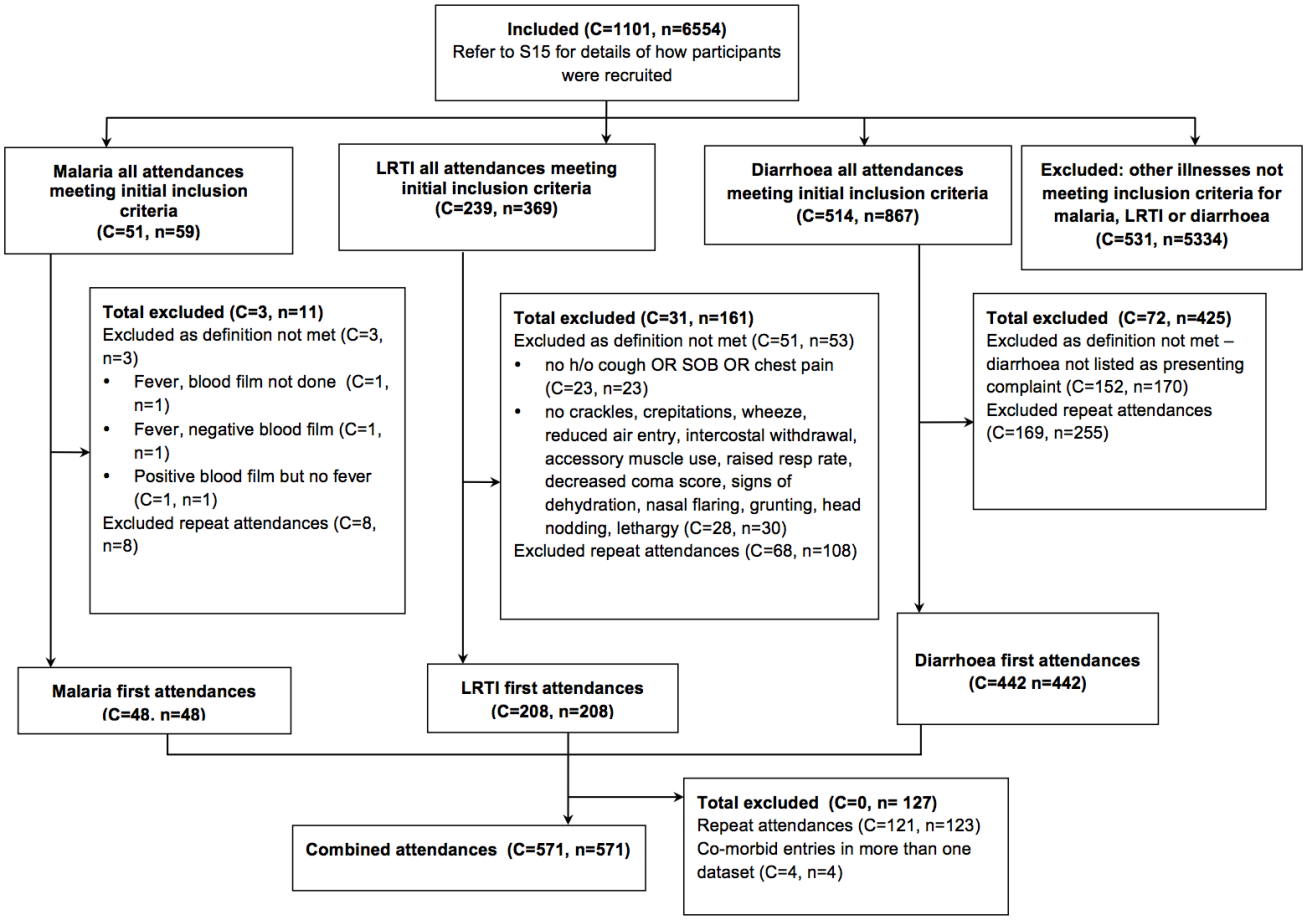
Flow Diagram of included participants and number of clinic attendances*. C = number of children, n = number of attendances. *The number of children at each stage of the flow diagram do not always add up as a child may have presented multiple times.

A total of 1101 children (44% of total population of children <5y in KW) were included with 6554 clinic attendances between 11 November 2009 and 02 April 2012. The mean clinic visit rate per child per year was 2.47 (95%CI[2.358, 2.583]). There were 48 first clinic presentations with malaria, 208 with LRTI and 442 with diarrhoeal disease by 571 children (Fig 2). There were four attendances with co-diagnoses—two with malaria and LRTI and two with LRTI and diarrhoeal disease. Study participant characteristics are detailed in Table 5.

**Table 5:** Study subjects and characteristics during first attendance with malaria, LRTI or diarrhoeal disease.

Variable	Number of children with available data	Number of all children included (%)	Number of all children with a delayed presentation (%)	Number of all children with a severe illness presentation (%)
**Male**	571	305 (53.4)	137 (53.3)	41 (54.0)
**Severe illness (severity score 2+)- all first attendances**	571	76 (13.3)	26 (5.9)	8 (44.4)
**Severe illness (severity score 2+)- malaria**	48	16 (33.3)	33 (12.8)	N/A
**Severe illness (severity score 2+)- LRTI**	208	63 (30.3)	28 (29.8)	N/A
**Severe illness (severity score 2+)- diarrhoea**	442	26 (5.9)	6 (3.1)	N/A
**Delayed presentation (symptoms 3 days+)- all first attendances**	571	257 (45.0)	N/A	33 (43.4)
**Delayed presentation (symptoms 3 days+)- malaria**	48	18 (37.5)	N/A	8 (50.0)
**Delayed presentation (symptoms 3 days+)- LRTI**	208	94 (45.2)	N/A	28 (44.4)
**Delayed presentation (symptoms 3 days+)- diarrhoea**	442	192 (43.4)	N/A	6 (23.1)
**Inhabitant of a ‘core village’**	571	261 (45.7)	85 (33.1)	26 (34.2)
**Only maternal child**	561	63 (11.2)	30 (12.0)	3 (4.2)
**Parents are monogamous**	277	24 (8.7)	10 (7.8)	4 (9.5)
**Parents not currently married**	571	173 (30.3)	79 (30.7)	25 (32.9)
**Child not living with mother**	571	57 (10.0)	29 (11.3)	10 (13.2)
**Death of mother**	571	3 (0.5)	1 (0.4)	0 (0.0)
**Death of maternal sibling**	571	34 (6.0)	12 (4.7)	9 (11.8)
**Mother ever attended English school**	570	99 (17.4)	46 (18.0)	14 (18.4)
		**Median (IQR)**		
**Distance to clinic (km)**	571	7.5 (11.6)	10.5 (11.8)	10.5 (10.2)
**Current age of mother (years)**	561	32 (10)	32 (11)	34 (10.5)
**Current age of father (years)**	457	49 (18)	49 (17)	50.5 (16)
**Number of maternal siblings**	561	4 (3)	4 (4)	4 (5)
**Number of paternal siblings**	460	7 (8.5)	8 (7)	7.5 (9)
**Age at first presentation (months)- all first attendances**	571	18 (20)	16 (18)	21.5 (31)
**Age at first presentation (months)- malaria**	48	40 (23.5)	45 (18)	37.5 (20.5)
**Age at first presentation (months)- LRTI**	208	20.5 (23)	18.5 (22)	20 (25)
**Age at first presentation (months)- diarrhoea**	442	15 (15)	14.5 (14)	12 (20)
**Number of wives of the child’s father**	277	2 (1)	2 (1)	2 (1)
**Birth order**	561	4 (4)	4 (4)	4 (5)

If a child presented more than once with different diseases only the first attendance is included, however the same child can appear in more than one individual disease dataset (shown in italics).

Using univariate analysis (see Tables 6–8), delayed presentation was associated with not being from a ‘core village’, and living further from the clinic. Severe illness was associated with the death of a sibling, not being from a ‘core village’, not being an only child, the child being older, mother being older, having more maternal siblings, and having a higher birth order.

**Table 6:** Univariate analysis of continuous independent variables.

Continuous independent variables	n	Mean difference prompt vs. delayed [95% CI]	t-test p-value	Mean difference non-severe vs. severe [95% CI]	t-test p-value
**Distance to clinic (km)**	571	-3.346 [-4.597, -2.096]	<0.0001	-1.482 [-3.353, 0.389]	0.120
**Child’s age (months)**	571	2.000 [-0.506, 4.505]	0.118	-3.917 [-7.580, -0.253]	0.036
**Mother’s age (years)**	561	0.240 [-0.915, 1.394]	0.684	-1.864 [-3.574, -0.154]	0.033

**Table 7:** Univariate analysis of ordered categorical independent variables.

Order categorical variable	n	Delayed vs. non-delayed, Kruskal-Wallis one way analysis chi-squared with ties	p- value	Severe vs. non-severe, Kruskal-Wallis one way analysis chi-squared with ties	p- value
**Number of maternal siblings**	561	0.372 with 1 d.f.	0.542	7.079 with 1 d.f.	0.008
**Birth order**	561	0.275 with 1 d.f.	0.600	5.004 with 1 d.f.	0.025

**Table 8:** Univariate analysis of dichotomous independent variables.

Dichotomous independent variables	Proportion prompt with variable (%)	Proportion delayed with variable (%)	Chi2 test p-value	Proportion non-severe with variable (%)	Proportion severe with variable (%)	Chi2 test p-value
**Severe illness**	43/314 (14)	33/257 (13)	0.765	n/a	n/a	n/a
**Delayed presentation**	n/a	n/a	n/a	224/495 (45)	33/76 (43)	0.765
**Male**	168/314 (54)	137/257 (53)	0.963	264/495 (53)	41/76 (54)	0.920
**Death of sibling**	22/314 (7)	12/257 (5)	0.240	25/495 (5)	9/76 (12)	0.020
**Death of mother**	2/314 (1)	1/257 (0.4)	0.684	3/495 (1)	0/76 (0)	0.496
**Mother attended English school**	53/314 (17)	46/256 (18)	0.733	85/494 (17)	14/76 (18)	0.795
**Parents are monogamous**	14/149 (9)	10/128 (8)	0.640	20/235 (9)	4/42 (10)	0.830
**From core village**	176/314 (56)	85/257 (33)	<0.0001	235/495 (47)	26/76 (34)	0.031
**Only child**	33/310 (11)	30/251 (12)	0.626	60/489 (12)	3/72 (4)	0.042

Using multivariate logistic regression, children from a ‘core village’ with access to free transport were significantly less likely to present delayed (OR 0.502, 95%CI[0.310, 0.814], p = 0.005) (Table 9). Children from villages with free regular transport were also less likely to present with severe illness (OR 0.557, 95%CI[0.325, 0.954], p = 0.033). Using Hosmer-Lemeshow goodness-of-fit test showed that the logistic regression models fit the data well (p = 0.354 for delayed presentation, p = 0.2723 for severe illness presentation).

**Table 9:** Multivariate regression of factors associated with delayed presentation and severe illness.

Presentation type	Identified variable using univariate analysis	Unadjusted		Adjusted for other variables significant in univariate analysis		Adjusted for other variables significant in univariate analysis and seasonality		Adjusted for other variables significant in univariate analysis and seasonality and year	
		OR (95% CI)	p-value	OR (95% CI)	p-value	OR (95% CI)	p-value	OR (95% CI)	p-value
**Delayed presentation**	‘Core village’	0.387, [0.275, 0.546]	<0.0001	0.527, [0.327, 0.849]	0.008	0.501, [0.309, 0.812]	0.005	0.502 [0.310, 0.814]	0.005
	Distance to clinic	1.059, [1.036, 1.083]	<0.0001	1.029, [0.997, 1.061]	0.073	1.023, [0.991, 1.056]	0.157	1.023 [0.991, 1.056]	0.159
**Severe illness**	Child’s age	1.016, [1.001, 1.031]	0.038	1.008, [0.986, 1.030]	0.495	1.008, [0.986, 1.031]	0.461	1.008 [0.986, 1.030]	0.484
	Only child	0.311, [0.095, 1.019]	0.054	0.545, [0.147, 2.020]	0.363	0.496, [0.133, 1.853]	0.297	0.503 [0.134, 1.881]	0.307
	Dead sibling	2.525, [1.130, 5.641]	0.024	2.096, [0.913, 4.813]	0.081	2.152, [0.928, 4.990]	0.074	2.174 [0.937, 5.041]	0.071
	Number of maternal siblings	1.159, [1.053, 1.275]	0.003	1.258, [0.716, 2.211]	0.425	1.129, [0.638, 1.996]	0.677	1.128 [0.639, 1.991]	0.678
	Mother’s age	1.039, [1.003, 1.077]	p = 0.034	0.993, [0.932, 1.058]	0.825	0.994, [0.932, 1.059]	0.850	0.995, [0.933, 1.060]	0.870
	‘Core village’	0.575, [0.347, 0.954]	0.032	0.589, [0.347, 1.002]	0.051	0.567, [0.332, 0.970]	0.038	0.557, [0.325, 0.954]	0.033
	Birth order	1.142, [1.038, 1.257]	0.006	0.894, [0.496, 1.608]	0.707	0.989, [0.547, 1.789]	0.970	0.990, [0.548, 1.788]	0.974

For the individual disease entities the association between illness severity and not being from a ‘core village’ was significant for the group of children presenting with an LRTI (OR 0.425, 95%CI[0.225, 0.800]], p = 0.008) (S9 Table).

For first presentations with diarrhoeal disease (S13 Table), children with severe illness (OR 0.314, 95%CI[0.116, 0.851], p = 0.023) were less likely to be delayed, as were those with previous death of a sibling (OR 0.297, 95%CI[0.092, 0.960], p = 0.042). Being from a core village decreased the risk of delayed presentation with borderline statistical significance (OR 0.559, [0.316, 0.988], p = 0.045). There was an increased risk of severe illness if there was a history of previous death of a sibling (OR 3.909, 95%CI[1.167, 123.096], p = 0.027). There was also borderline decreased risk of severe illness in those with delayed presentation, (OR 0.378 [0.144, 0.992], p = 0.048).

With regards to the sensitivity analysis, 82 attendances with clinical data and no recorded diagnosis or an ICD-10 code of ‘R69 unknown diagnosis’ were identified. We examined clinical data for each attendance to assign them to each disease category where they met the crtiteria. The data analysis was repeated with these attendances, again removing repeat attendances. Although, effect sizes and p values changed slightly as expected with more ‘crude’ data, the overall results were not affected providing evidence for limited bias and support of our study findings.

## Discussion

To our knowledge this is the first study that investigates factors affecting delay and severity of presentations to a rural African primary healthcare centre using an electronic medical record system integrated within a demographic surveillance system.

Our results show that delayed presentation is a significant problem in this setting with children coming to the clinic 3 or more days after the onset of symptoms in around 45% of presentations. However, comparable data from other low- and high-income settings is limited. In 2000, African leaders signed up to the Abuja declaration that by 2005 at least 60% of those with malaria will have access to affordable and appropriate treatment within 24 hours of onset of symptoms [59]. A survey in The Gambia found that 48% of children, compared to only 21% in our study, received an appropriate anti-malarial drug within 24 hours of the onset of symptoms [60]. This discrepancy may be due to differences in access to transport, socioeconomic status and/or level of education. There is a wide variation in percentages receiving appropriate anti-malarials within 24 hours amongst the literature- 13% of <5y[61], 68.8%[44], 35%[41], 11%[62], 61%[45]. Many of these studies consider home treatment and treatment from sources other than a health facility, which is why the figures may be higher. As our current study highlights, access to transport may be a significant contributor to delay in accessing health care. KW has one of the worst accesses to transport and main roads within in The Gambia. Most other health centres in The Gambia are located close to a main paved road[47]. Furthermore, KW is one of the most deprived areas in The Gambia[50]. In line with socioeconomic differences, educational levels within KW for women of childbearing age are lower than levels in urban areas of The Gambia[50].

We were able to demonstrate with this study that access to transport rather than distance was significantly associated with delay in presentation and severe illness at presentation supporting suggestions of a previous systematic review that there is no definite evidence of an association between distance *per se* to health services and that distance may not represent time travelled or availability of transport [34] (Table 2). When all diseases were combined, those living in the ‘core villages’ with free regular transport were statistically significantly more likely to present promptly and present with less severe illness. Other villages, even including those villages less than 10km away from Keneba (Fig 1) are reliant on infrequently passing vehicles, bush taxis or walking long distances. Apart from access to transport alone, transport cost is also a known barrier to children accessing care and needs to be further investigated in this setting [42, 33].

Interestingly, less than 6% of those with diarrhoeal disease had severe illness compared to a third of those with LRTI and malaria. Reasons for this may be lack of recognition of early stages of malaria and LRTI, more rapid progression or use of alternative treatment sources initially in these conditions. A study in Kenya showed that care was sought more often for diarrhoea than for coughing [17]. This suggests a need to focus on educational intervention of guardians to recognise features of severity of respiratory and malarial diseases.

We had hypothesised that younger aged mothers may lack the knowledge and experience to manage child illness and this may affect access. Death of the child’s mother or being separated from the mother may influence access as the child will lack the usual primary caregiver. However neither maternal age nor death of the mother were significantly associated with either severe illness or delayed presentation.

Death of an older sibling has previously been associated with increased mortality[26]. From our data there is an indication that children with a death of a maternal sibling may be more likely to present with severe illness. However, the association was not statistically significant for combined clinic visits (OR 2.152, 95%CI[0.928, 4.990], p = 0.074) but was significant for those with diarrhoeal disease (OR 3.909, 95%CI[1.167, 13.096]], p = 0.027) (S13 Table). The wide confidence interval reflects the small number of sibling deaths in this cohort (n = 34 for combined first attendances).

In patriarchal societies there can be a preference for male children to receive better care [63] but evidence of the influence of gender on access to health is conflicting (Table 2). Furthermore parents may be more likely to bring younger children promptly. Again neither age nor gender seemed to have influenced access to health as measured by illness severity or delay in presentation.

Previous studies that used delayed presentation as an outcome, only focused on malaria presentations and confirmation of diagnosis by blood film was often lacking [62, 44, 61, 41, 45, 64, 65]. This study, however, addressed delayed presentation for the three main causes of morbidity in the region, LRTI, diarrhoeal illness and malaria and the latter was confirmed by blood film analysis in all cases.

We recognise that there are limitations to the current study. Due to small numbers for each individual disease entity, focus was directed at combined attendances and co-diagnoses were grouped as a single presentation to avoid duplication. The authors also acknowledge the same child may be included in more than one separate disease entity dataset but each child was only presented once in the combined dataset (Fig 2).

There were only small numbers of presentations meeting our criteria as ‘severe’, this may have affected the ability to detect variables associated with severe illness. The definitions used for delayed presentation and severe illness are unique to this study. We tried to corroborate different elements of the history and examination to determine a severe illness. It could be argued that a child presenting within 3 days with malaria is still a delayed presentation, likewise for a child with mild diarrhoea it might be reasonable to wait longer than 3 days before presenting.

Although all children with a positive malaria film were included as a diagnosis of malaria, only children with a physician made diagnosis were included for LRTI and diarrhoeal disease. Some children, who possibly had diarrhea or LRTI, but without a diagnosis may have been missed. Retrospective diagnosis from clinical notes was not made, as consistency of diagnostic inclusion within the set parameters of this study could not be guaranteed.

The cohort of children studied was recruited as part of a clinical study and may differ from other children presenting. However, clinic attendance per child, disease patterns of clinic presentations and socio-demographic background of the study participants is comparable to the general paediatric population resident in KW[50]. The authors are not aware of any published data available on clinic attendance per child per year in a similar cohort in this setting. The wide variation of clinic presentations in KW may suggests differences in health status as well as more limited access to health care for some children and led to the conduction of this study.

Families living close to the healthcare centre may have been more exposed to westernized healthcare management and have a higher healthcare awareness in general. Research studies in Keneba were started in the 1950’s [51] and children under 3y from the ‘core villages’ attend routine child welfare clinics. However, free healthcare is provided for the whole of KW region and all villages are included in the KWDSS and many families outside the ‘core villages’ have been involved in ongoing research studies raising healthcare awareness in the general population in KW[50]. Also, we only included self-referred clinic visits and excluded all child welfare and research clinic presentations. Patients with milder disease courses may not have come at all to the clinic if they live far away, as demonstrated by Moisi and colleagues in Kenya[31], we were only able to assess children who presented.

Delayed presentations were defined as a delay of 3 days or more based on guardians’ reports. Parental recall has its own limitations with intra-observer variation and also differences between interviewers (three different physicians were employed at MRC Keneba during the study period) [66]. The length of symptomology may have been exaggerated by the guardian after travelling from far away. However, duration of symptoms can only be established on history taking and we have no evidence for any biased reporting by those living out with ‘core villages’.

In this study there were factors which may have influenced access that could not readily be assessed such as socio-economic status, the role of the extended family, transport methods taken, travel time and cost, usage of traditional medicine, and maternal autonomy with regards to decision making and access to finances. Qualitative studies are needed to explore these themes further.

## Conclusions

Using high quality data from a well-described study population, this study has shown that delayed presentation to primary healthcare and severe illness at presentation is associated with poor access to transport rather than distance to the health facility. It suggests access to healthcare remains a problem even when healthcare is provided free of charge. Many children are presenting late which may affect the success of treatment and associated outcomes. Future public health interventions should focus on improving transport availability to maximise access to primary healthcare in low-income settings.

## Supporting Information

S1 File Cohort recruitment criteria. (DOCX)

S2 File Literature search strategy. (DOCX)

S3 File PRISMA flow diagram. (DOCX)

S4 File Literature review summary. (DOCX)

S1 Table How PAWS score was calculated. (DOCX)

S2 Table Attendances with malaria- results of univariate analysis of continuous independent variables. (DOCX)

S3 Table Attendances with malaria- results of univariate analysis of ordered categorical independent variables. (DOCX)

S4 Table Attendances with malaria- results of univariate analysis of dichotomous independent variables. (DOCX)

S5 Table Attendances with malaria- results of multivariate logistic regression for factors identified as significant in univariate regression analysis. (DOCX)

S6 Table Attendances with LRTI- results of univariate analysis of continuous independent variables. (DOCX)

S7 Table Attendances with LRTI- results of univariate analysis of ordered categorical independent variables. (DOCX)

S8 Table Attendances with LRTI- results of univariate analysis of dichotomous independent variables. (DOCX)

S9 Table Attendances with LRTI- results of multivariate logistic regression for factors identified as significant in univariate regression analysis. (DOCX)

S10 Table Attendances with diarrhoeal disease- results of univariate analysis of continuous independent variables. (DOCX)

S11 Table Attendances with diarrhoeal disease- results of univariate analysis of ordered categorical independent variables. (DOCX)

S12 Table Attendances with diarrhoeal disease- results of univariate analysis of dichotomous independent variables. (DOCX)

S13 Table Attendances with diarrhoeal disease- results of multivariate logistic regression for factors identified as significant in univariate regression analysis. (DOCX)

S14 Table All clinic attendances. Sheet 1: data from all clinic attendances with data from DSS included. Sheet 2: explanatory Data Dictionary.
(XLS)

S15 Table Codes on KEMRes. (DOCX)
