# Supplementary material for: Factors Affecting Access to Healthcare: An Observational Study of Children under 5 Years of Age Presenting to a Rural Gambian Primary Healthcare Centre
Source: PLoS One. 2016 Jun 23;11(6):e0157790. doi: 10.1371/journal.pone.0157790 (PMC4919103; doi:10.1371/journal.pone.0157790)
Supplement: S1 File — (DOCX) [file pone.0157790.s001.docx]

**S15 Description of recruitment of cohort studied**

The cohort of children described in the current study were participants of a randomised controlled trial of lipid-based micronutrients (ISRCTN73571031).

All KW resident children aged 6m to 60m presenting with an acute illness to the Keneba primary health care centre were eligible for inclusion in the study. Children enrolled in another research study, having known haemoglobinopathies or severe malnutrition were excluded. Children with severe malnutrition as defined by the WHO were treated at the MRC Keneba nutritional rehabilitation centre and then became eligible for enrolment at a subsequent uncalled presentation after successful management. Children and infants called for child welfare clinics, clinical follow-up appointments and immunizations were excluded unless they presented with an acute illness. Children were eligible for enrolment at a subsequent uncalled presentation.

2743 primary health care clinic presentations were assessed for eligibility. 1642 children were either not eligible (54 children were called for child welfare clinic or research purposes rather than self-presented, 130 children had an excluded diagnoses, 1015 were not residents of Kiang West, (the study area), 55 children were enrolled in other projects, 388 were not approached or consent was not given.

1101 children were recruited between Dec 2009 and June 2011 and were included in the present study for all the clinic attendances documented in KEMReS before and after this recruitment period for the clinical trial.
