# Supplementary material for: Factors Affecting Access to Healthcare: An Observational Study of Children under 5 Years of Age Presenting to a Rural Gambian Primary Healthcare Centre
Source: PLoS One. 2016 Jun 23;11(6):e0157790. doi: 10.1371/journal.pone.0157790 (PMC4919103; doi:10.1371/journal.pone.0157790)
Supplement: S2 File — (DOCX) [file pone.0157790.s002.docx]

# PlosONE Literature review Search Strategy

**Medline (search date 30.01.16)**

1. Health Services Accessibility = 55731

2. “health care seeking” = 709

3. Health$5 ADJ5 utili#ation = 17,860

4. health* ADJ5 access* = 72,039

5. Infant mortality/ OR Child mortality/ = 26,947

6. Delay* presentation = 1463

7. (illness or disease or presentation) ADJ1 sever$3 = 44,055

8. Distance.mp = 166,157

9. Time factors/ = 1,044,017

10. cost.mp = 366,171

11. Health Knowledge, attitudes and Practice/ = 80,469

12. SocioEconomic Factors/ = 122,719

13. “Patient Acceptance of Health Care”/ = 335,22

14. transport.mp = 485,063

15. Africa*.mp = 238,223

16. p?ediatric.mp OR child*.mp OR Child/ or Infant/ = 2,203,573

17. [1] or [2] or [3] or [4] = 87,912

18. [3] or [5] or [6] or [7]= 89,891

19. [8] or [9] or [10] or [11] or [12] or [13] or [14] = 1,751,242

20. [15] and [16] and [17] and [18] and [19] = 197

21. Limit [20] to English language = 191

**Embase (search date 30.01.16)**

1. ‘health’/exp OR health AND services AND accessibility = 4831
2. health and care and seeking = 23,699
3. health and (care or service) and (utilisation or utilization) = 126995
4. ‘africa’ = 235,153
5. ‘child’.de or child = 2,178,153
6. health and care and access = 118,196
7. ‘child mortality’.de or ‘child mortality’ = 10,612
8. delay* and presentation = 21,808
9. ‘disease severity’.de or ‘disease severity’ = 417,943
10. socio economic status = 9,587
11. health knowledge attitudes practice.de or health knowledge attitudes practice = 85,238
12. ‘time’.de= 359,557
13. ‘cost’ = 634, 194
14. patient acceptance of health care = 52,112
15. transport = 644,754
16. distance = 199,960

17. [1] or [2] or [3] or [6] = 248,637

18. [7] or [8] or [9] or [3] = 696,618

19. [10] or [11] or [12] or [13] or [14] or [15] or [16] = 1,927,459

20. [17] and [18] and [19] and [4] and [5] = 361

21. Limit [20] to English Language = 357
