# Supplementary material for: Factors Affecting Access to Healthcare: An Observational Study of Children under 5 Years of Age Presenting to a Rural Gambian Primary Healthcare Centre
Source: PLoS One. 2016 Jun 23;11(6):e0157790. doi: 10.1371/journal.pone.0157790 (PMC4919103; doi:10.1371/journal.pone.0157790)
Supplement: S3 File — (DOCX) [file pone.0157790.s003.docx]

**PRISMA Flow Diagram**

Records identified through database searching (Medline and Embase) = 548

Additional records identified through other sources = 21

Identification

Records excluded on title = 396

Full‐text articles excluded = 38

Full‐text articles assessed for eligibility = 77

Studies included in

qualitative synthesis

= 39 papers

Records excluded on abstract = 50

Records screened = 127

Records after duplicates removed= 523

Included

Eligibility

Screening
