# Supplementary material for: Factors Affecting Access to Healthcare: An Observational Study of Children under 5 Years of Age Presenting to a Rural Gambian Primary Healthcare Centre
Source: PLoS One. 2016 Jun 23;11(6):e0157790. doi: 10.1371/journal.pone.0157790 (PMC4919103; doi:10.1371/journal.pone.0157790)
Supplement: S4 File — (DOCX) [file pone.0157790.s004.docx]

**S18 Table**

**Summary of studies identified through the literature review.**

| **Paper** | **Setting** | **Outcome measure** | **Description and limitations** | **Explanatory variables** |
| --- | --- | --- | --- | --- |
| **Ahonsi 1995** | Nigeria | Child mortality | - Based on DHS data - Only univariate analysis | - Mother <20 yrs associated with increased mortality - Increased mortality with reduced general health services spatial density - Increased mortality with reduced birth spacing |
| **Alegana 2012** | Namibia | Healthcare utilization | - Treatment seeking for fever for children <5y, using survey data - Assumptions of mode of transport taken and choice of health facility - Travel time used as a continuous variable | - Likelihood of utilization remained high up to 3 hrs travel time then decreased steadily |
| **Armstrong Schellenberg 2008** | Rural Tanzania | Infant mortality, healthcare utilization for illness in last 2/52 | - Household survey, 21,000 - Distance measured as straight-line binary variable - No multivariate regression | - No difference in male and female infant mortality rates; Infant mortality higher for teenage mothers - More appropriate care-seeking if closer to health facility - Increased infant mortality if >5km from health facility - Increased risk being a twin or first child born or reduced birth spacing - Increased risk for some ethnic groups - Increased risk if mother had no formal education; Increased risk if death of mother - Increased risk if death of older sibling - Increased risk if <1y born during rainy season |
| **Becher 2004** | Rural Burkina Faso | Child mortality | - Did not do multivariate analysis to account for confounding | - Increased risk if mother <18 - Increased risk in children <1y living >10km away - Increased risk being a twin or first child born or reduced birth spacing - Increased risk for some ethnic groups - Increased risk if death of mother; increased risk if death of older sibling - Increased risk if <1y born during rainy season |
| **Buor 2003** | rural Ghana | Utilisation of health care in last 3 illness episodes | - 400 household interviews, not restricted to children - Did multivariate regression; - Doesn't specify how distance was measured, used as ordered categorical variable | - Lesser effect of transport cost on utilisation - Close correlation between distance and travel time, both associated with utilization |
| **Burgert 2011** | Rural Kenya | Health care utilization | - Rate of sick-child visits per population for all acute illnesses | - Reduced risk if from small ethnic group, decreased risk if better economic status - More likely to attend if symptoms of severe illness |
| **Burton 2011** | Rural Kenya | Health care utilization for diarrhoea, fever or ARI | - Household survey, 1679 <5y children - Relied on parental recall of fever, diarrhoea, ARI over last 2/52 and pneumonia over last 12/12. - Reported visits not validated by checking hospital records | - Increased utilisation with implementation of high quality free health care |
| **Byass 2008** | rural Ethiopia | Child mortality | - Birth cohort from 1987 study, 1884 livebirths - Distance to health facilty assessed as <5km or >5km | - Distance to health facility <5km vs >5km not significant in multivariate analysis - Increased risk with parity >5 - Increased risk with chronic illness in mother - Increased risk if 2+ dead siblings |
| **Feikin 2009** | rural Kenya | Health care utilisation, distance travelled to clinic | - 3501 clinic visits by 2432 children - Illness severity assessed on diagnosis only- malaria with convulsions, pneumonia with convulsions - Straight-line distance used | - Younger children travelled further than older children - Rate of clinic visits decreased with increasing distance to clinic - Higher SE status associated with more clinic visits - Higher maternal education status associated with more clinic visits - Children with severe illness travelled further than those with non-severe illness |
| **Fosu 1994** | 6 African countries | Healthcare utilization | - DSS- childhood illness in last 2/52 and whether care sought from healthcare facility | - Mixed effect of child's age - Higher utilization by younger mothers - More likely to attend if higher SE status by household possessions - Children with more severe pneumonia more likely to be taken to health facility than those with less severe pneumonia |
| **Franckel 2009** | Senegal | Probablitiy of seeking malaria treatment from health facility within 48 hrs of symptom onset; 902 illness febrile episodes from household survey | - From DSS survey and household survey - 902 children with suspected malaria - Reference period of 7-18 days asked about febrile illnesses - Not confirmed malaria; relied on parental recall - Distance measured as continuous variable, not clear if was straight-line measurement. | - The delay resorting to the health centre was longer as the child got older with a threshold starting from age 7 (OR=0·52; p=0·036) - Child's sex did not predict prompt treatment - The distance to health facilities strongly influenced the probability of consulting a health centre- figures not given. - Visits to health centre decreased regularly as the number of children below 6 living in the household grew. - The Peul and Wolof, two ethnic groups not traditionally established in this area, resorted more promptly (OR=0·28) to the dispensary then the Sereer. SE status by goods owned not significant. Level of parental education did not affect chance of prompt treatment. - The child was brought quickly to the dispensary when he presented severe signs of malaria– high fever, vomiting, asthenia, poor appetite (OR=2·13; p=0·002). - Visits to health centre decreased regularly as the number of children below 6 living in the household grew. |
| **Getahun 2010** | Ethiopia | Delayed presentation with malaria in children <5y | - Case-control study- children presenting within and post 24hr after symptom onset with confirmed malaria - 155 cases and 155 controls - Multivariate regression done | - More likely to be delayed if complained about higher cost of transportation - Living >3km from health centre associated with delay - Children of monogamous marriages more likely to be delayed OR 3.41(1.39.8.34) - No h/o child death more likely to be delayed- OR3.50 (1.82, 6.42) |
| **Gething 2004** | Kenya | Healthcare utilization | - Measured straight line distance | - Steady decline in utilization within catchment area up to 6km |
| **Holtz 2003** | Malawi | Prompt effective treatment of malaria; health facility attendance | - Household survey recalling febrile episodes in last 14/7 - 292 children with recent febrile illness | - Decreased utilization if possession of fewer resources - Religion affected utilization- mixed effect - Decreased utilization with low mother education - Decreased utilization if fewer illness episodes |
| **Justesen 2000** | Malawi, Tanzania & Zambia | Child mortality for singletons and twins | - DHS data from Malawi, Tanzania & Zambia | - Participants were willing to travel further to reach higher order facilities |
| **Kadobera 2012** | rural Tanzania | Infant and child mortality | - Used network distance (physical path or road followed) rather than straight-line distance, analysed as dichotamous variable <5km and >5km - Did not have information on method of transport - Did multivariate analysis | - Higher age had lower mortality - Higher mortality for males (HR 1.16[1.01-1.35]) - Increased mortality for >5km (HR 1.17 [1.02,1.38]) - Increased mortality with multiple birth and bigger family size but decreased mortality with higher maternal parity - Increased mortality if death of mother - Increased mortality if death of sibling |
| **Kahabuka 2013** | Tanzania | Prompt (same or next day) treatment with anti-malarial; treatment with ORS for diarrhoea- included treatment from pharmacy. | - National household survey; based on mother's report of febrile illness in 2/52 prior to survey | - The proportion of children who received prompt treatment with any antimalarial, i.e. during the same or next day, was found to be higher in children of mothers with only one child compared to those with 2 or more children below five years (OR: 2.0 (1.4–2.8)) - Higher proportion received ORS for diarrhoea if only one child <5y vs. those with 2 or more children below five years (OR: 1.4 (1.1–1.9)) - The proportion of children who received prompt treatment with any antimalarial, i.e. during the same or next day, was found to be higher among children in urban compare to rural areas (OR: 1.5 (1.0–2.4)). - The proportion of children who received ORS or HRS was also higher among caretakers with higher compared to lower SES (OR: 1.8 (1.2–2.8)). - The proportion of children who received prompt treatment with any antimalarial, i.e. during the same or next day, was found to be higher among children of mothers with primary or higher compared to no education (OR: 1.3 (0.9–2.0)). - Parental education did not influence ORS use for diarrhoea. |
| **Kazembe 2006** | Malawi | Hospitalisation and mortality | - Assessed distance as dichotomous variable, not transport | - Increased mortality rate with distance >5km vs <5km - Rural location less likely to get prompt effective treatment |
| **Kenny 2015** | rural Liberia | Healthcare utilization and choice of provider | - Distance categorised into 4 quartiles; GPS measured road distance - Care seeking for ARI, fever and diarrhoeal illness in last 2 weeks - Used multivariate regression | - Children in further quartiles less likely to seek care for fever. Children in fartherest quartiles more likely to seek care from informal provider for ARI and diarrhoeal illness. - Mortality twice as high for twins than singletons. - Increase mortality with short birth spacing for both singleton and twin pregnancies. - Increased mortality with decreased maternal education |
| **Manongi 2014** | rural Tanzania | Hospital admission rates, Inpatient case fatality rates and child mortality rates | - Children admitted to hospital with fever - Travel time as ordered categorical variable | - More likely to have travelled for longer if child male. - Travel time >3hr associated with decreased utilization and increased mortality rate, longer illness duration, shorter time from admission to death |
| **Materia 1993** | Ethiopia | Healthcare utilization of under 5 clinic (preventive and curative) | - Only univariate analysis at <0.05 - Did not specify how distance was measured - Statistics not given | - No association with distance to clinic |
| **Moisi 2011** | Kenya | Hospitalistion with all causes, pneumonia and meningitis, hospital mortality rates and access to care ratios (community vs. hospital mortality rates) for <5y | - Used hospital records and DSS - Travel times for pedestrian and vehicles calculated using GPS - Travel time measured as a continuous variable | - Lower hospitalisation rates for females; males experienced reduced distance decay effect - Hospitalisation rates decreased with increasing travel time; hospital mortality rates increased with travel time - Lowest quintile SE status higher mortality, mortality reduced with decreased poverty |
| **Muller 2003** | rural Burkina Faso | Healthcare utilization | - Cohort of children followed for 6/12, interviewed on treatment seeking for episodes of febrile illness - Did not do multivariate analysis | - More likely to seek care if health centre in village of residence or hospital nearby |
| **Nasrin 2013** | rural Gambia (and others) | Healthcare utilization and management of diarrhoea | - Household surveys | - No association with mother's ethnicity - No association with mother's education |
| **O'Meara 2009** | Kenya | Rates of hospitalisation with malaria | - Rates of hospitalisation with malaria related to walking time to nearest primary care facility - Walking time analysed as a continuous variable - Did not adjust for confounders | - Increased rates of hospitalisation with increasing walking time to primary care clinic |
| **O'Reilly 2012** | rural Kenya | Child mortality amongst children hospitalised with diarrhoea | - 1146 children enrolled, 107 died | - No association with gender - Children who died presented with longer duration of symptoms |
| **Odu 2015** | Nigeria | Healthcare utilization for severe fever in last 1/12 | - Household surveys | - Decreased utilization if no community healthcare facility - More likely to seek care for fevers >=38.5 - No association of fever duration and visiting a health centre |
| **Parmar 2014** | Burkina Faso | Health care utilization | - Household survey; not limited to children - Distance <5km or >5km, economic status poor or rich - Comparison of those with and without insurance | - Effect of insurance scheme- did not reduce effect of distance. Higher wealth index assoc with seeking care from licensed providers. - Of children with MSD those with lethargy were more likely to visit a health centre; children taken to licensed provider more likely to have fever or lethargy; other signs of severe illness- decreased urinartion, excessive thirst, dry mouth, wrinkled skin, bloody stool, rice water stool, >6 stools per day, vomiting >3/day were not associated with seeking licensed care |
| **Rutherford 2010** | SSA | Child mortality | - Systematic review of studies looking at access- distance, social factors effect on child mortality - Did not do multivariate analysis | - Mixed findings regarding association of distance with child mortality - Social factors such as support available for the mother found to affect child mortality |
| **Rutherford 2009** | Gambia | Child mortality | - Case-control study - Distance measured by GPS, binary variable; also measured as travel time binary variable - Used multivariate regression | - Cost of transport not significant - Distance (GPS, binary variable) and travel time (binary variable, by mode of transport) not significant - Children whose primary caregivers cared for other children were less likely to die than those whose primary caregivers cared for no other children, regardless of the number of children cared for: (1–3 other children: OR: 0.2; 95% CI: 0.1–0.5; 4 or more other children: OR: 0.2; 95% CI: 0.1− 0.4) - Non-traditional variables, including indicators of social support for the primary caregiver, his/her degree of financial autonomy and his/her source of revenue for health-care expenses, were significantly associated with child death - Rural dwellers had a significantly greater likelihood of death than urban dwellers (OR: 4.9; 95% confidence interval, CI: 1.2–20.2). |
| **Sartorius 2011** | South Africa | Child Mortality | - 46,675 children resident between 1992-1997, 565 deaths - Did multivariate analysis - Network distance as dichotomous variable used | - Decreased mortality with increasing child age - No association with distance to clinic - Decreased utilization if no clean water, insufficient food - Decreased utilization if parental low education - Decreased utilization if fever <5/7 |
| **Schoeps 2011** | rural Burkina Faso | Child mortality | - Distance measured as walking travel time in wet and dry season - Adjusted for confounders- sex, birth order, mother age, twin birth, year of birth, death of older sibling, death of mother, birth spacing, religion, ethnicity - No ambulances or public transport available. | - The estimated 5-year survival for children living less than 30 minutes from a health facility was 87.7% (95% confidence interval (CI): 86.9, 88.4), while it was only 82.1% (95% CI: 81.1, 83.1) for those with travel times over 120 minutes. CI: 81.1, 83.1) for those with travel times over 120 minutes. The mortality hazard increased by a factor of 1.12 (95% CI: 1.07, 1.17) for each additional hour of walking time, which results in a doubled risk for children who had to travel more than 6 hours to the closest health facility. - Children from poorest households 20-30% less likely to seek care; ethnicity influenced care seeking. - Carer perceived illness severity influenced likelihood to seek care; more likely to seek care for diarrhoea than cough |
| **Sodemann 2006** | Guinea-Bissau | Child mortality | - 1572 children who attended emergency clinic, 30 day mortality assessed | - Increased mortality with younger mother - No association between parental education and child mortality - No association between death of sibling and child mortality |
| **Taffa 2005** | Urban Kenya | Healthcare utilization | - Household survey, interviewed 999 about illnesses in last 2 wks - Place of care included drug shops - Multivariate regression analysis done | - Less likely to seek care for children aged 4-5 - Mothers >35 less likely to take children to seek care |
| **Tipke 2009** | Burkina Faso | Care seeking and treatment of malaria (includes home treatment) | - Household survey, recall malaria episodes in last 1/12 - 239 out of 802 children surveyed had episode - Only univariate analysis - Malaria episodes not clinically confirmed - Distance analysed as a dichotomous variable | - Age of child not associated with treatment within 24 hrs - In localities with a health facility compared to localities without one about twice as many children were taken to a medical professional first (27% vs. 13%, p =0.005). - Distance to health facility not associated with malaria treatment within 24 hrs - Visiting a trained healthcare provider was assoc with longer travel times and higher direct costs - Utilization with illness severity not significant - Mother education did not affect utilization - Children from poorest households and >12/12 less likely to seek care - No effect of rural vs. urban location |
| **Ustrup 2014** | Malawi | Child healthcare utilization | - Household survey | - Child's gender did not affect utilization - Having insurance increased utilization |
| **Van den Broeck 1996** | rural Zaire | Child mortality | - Multivariate regression done - 30 mths survey of 776 children - Distance analysed as a dichotomous variable | - Increased risk if >5km to dispensary |
| **Wagstaff 2000** | Multiple countries (Côte d’Ivoire, Ghana, South Africa ) | Infant and child mortality | - Survey data | - Increased mortality if 4+ children <5y in household - Increased mortality if Mozambique origin - Increased mortality with death of mother, death of father; - Increased mortality with death of other household members - Increased mortality in winter season |
| **Zoungrana 2014** | Burkina Faso | Severe malaria in children <5y | - Cross-sectional study of 510 children presenting with confirmed malaria - Multivariate regression done - WHO definitions of severe malaria used - Distance and travel time as binary variables | - Not associated with child age - Not associated with child gender - Age of caregiver not associated - Distance and travel time not associated with severe illness - Children who died had longer symptom duration before presentation |
