# Supplementary material for: Factors Affecting Access to Healthcare: An Observational Study of Children under 5 Years of Age Presenting to a Rural Gambian Primary Healthcare Centre
Source: PLoS One. 2016 Jun 23;11(6):e0157790. doi: 10.1371/journal.pone.0157790 (PMC4919103; doi:10.1371/journal.pone.0157790)
Supplement: S1 Table — (DOCX) [file pone.0157790.s005.docx]

**S1 Table**

How PAWS score was calculated.^1^

|  | **Observation** | **Age group (months)** | **0 points** | **1 point** | **2 points** | **3 points** |
| --- | --- | --- | --- | --- | --- | --- |
| 1 | **Respiratory rate** (breaths per min) | 0-11 | 21-49 | 50-59 | <21  60-69 | 70+ |
|  |  | 12-23 | 16-44 | 45-54 | <16  55-64 | 65+ |
|  |  | 24-59 | 16-39 | 40-49 | <16  50-59 | 60+ |
|  |  | 60+ | 11-34 | 35-44 | <11  45-54 | 55+ |
| 2 | **Heart rate** (beats per min) | 0-11 | 91-179 | 71-90  180-199 | 51-70  200-219 | <51  220+ |
|  |  | 12-23 | 81-169 | 61-80  170-189 | 41-60  190-209 | <41  210+ |
|  |  | 24-59 | 76-159 | 56-75  160-179 | 36-55  180-199 | <36  200+ |
|  |  | 60+ | 61-139 | 41-60  140-159 | 21-40  160-179 | <21  180+ |
| 3 | **Work of breathing** |  | Normal |  | Chest indrawing OR accessory muscle use | Chest indrawing AND accessory muscle use |
| 4 | **Oxygen saturation levels** (%) |  | 93+ | 90-92 | 85-89 | <85 |
| 5 | **Body temperature** (°c) |  | 36-37.9 | 38-38.9 | 35-35.9  39+ | <35 |
| 6 | **Capillary refill time** (sec) |  | 0-3 |  | 4-6 | >6 |
| 7 | **Coma score** |  | 15/15 | 9-14/15 | 4-8/15 | 3/15 |

##### 1. For each of the 7 observations, points are given out of a maximum of 3. The total PAWS score is the combined points. If this is 3 or above this gives one point to the overall severity score.
