# Supplementary material for: Factors Affecting Access to Healthcare: An Observational Study of Children under 5 Years of Age Presenting to a Rural Gambian Primary Healthcare Centre
Source: PLoS One. 2016 Jun 23;11(6):e0157790. doi: 10.1371/journal.pone.0157790 (PMC4919103; doi:10.1371/journal.pone.0157790)
Supplement: S2 Table — (DOCX) [file pone.0157790.s006.docx]

**S2 Table**

Attendances with malaria- results of univariate analysis of continuous independent variables.

| **Continuous independent variables** | **n** | **Mean difference prompt vs. delayed [95% CI]** | **t-test**  **p-value** | **Mean difference non-severe vs. severe [95% CI]** | **t-test**  **p-value** |
| --- | --- | --- | --- | --- | --- |
| **Distance to clinic (km)** | 48 | -1.158 [-5.018, 2.702] | 0.549 | -0.353 [-4.331, 3.625] | 0.859 |
| **Child’s age (months)** | 48 | -5.478  [-14.985, 4.030] | 0.252 | 5.219 [-4.565, 15.003] | 0.289 |
| **Mother’s age (years)** | 45 | -0.586 [-4.171, 2.999] | 0.743 | -3.244 [-6.819, 0.331] | 0.074 |
