# Supplementary material for: Factors Affecting Access to Healthcare: An Observational Study of Children under 5 Years of Age Presenting to a Rural Gambian Primary Healthcare Centre
Source: PLoS One. 2016 Jun 23;11(6):e0157790. doi: 10.1371/journal.pone.0157790 (PMC4919103; doi:10.1371/journal.pone.0157790)
Supplement: S3 Table — (DOCX) [file pone.0157790.s007.docx]

**S3 Table**

**Attendances with malaria- results of univariate analysis of ordered categorical independent variables.**

| **Order categorical variable** | **n** | **Delayed vs. non-delayed**  **Kruskal-Wallis one way analysis**  **Chi-squared with ties** | **p- value** | **Severe vs. non-severe**  **Kruskal-Wallis one way analysis Chi-squared with ties** | **p- value** |
| --- | --- | --- | --- | --- | --- |
| **Number of maternal siblings** | 45 | 0.001 with 1 d.f. | 0.971 | 6.119 with 1 d.f. | 0.013 |
| **Birth order** | 45 | 0.041 with 1 d.f. | 0.839 | 4.893 with 1 d.f. | 0.027 |
