# Supplementary material for: Factors Affecting Access to Healthcare: An Observational Study of Children under 5 Years of Age Presenting to a Rural Gambian Primary Healthcare Centre
Source: PLoS One. 2016 Jun 23;11(6):e0157790. doi: 10.1371/journal.pone.0157790 (PMC4919103; doi:10.1371/journal.pone.0157790)
Supplement: S4 Table — (DOCX) [file pone.0157790.s008.docx]

**S4 Table**

**Attendances with malaria- results of univariate analysis of dichotomous independent variables**

| **Dichotomous independent variables** | **Proportion prompt with variable (%)** | **Proportion delayed with variable (%)** | **Fisher’s exact test**  **p-value** | **Proportion non-severe with variable (%)** | **Proportion severe with variable (%)** | **Fisher’s exact test**  **p-value** |
| --- | --- | --- | --- | --- | --- | --- |
| **Severe illness** | 8/30  (26.67) | 8/18  (44.44) | 0.226 | N/A | N/A | N/A |
| **Delayed presentation** | N/A | N/A | N/A | 10/32  (31.25) | 8/16  (50.00) | 0.226 |
| **Male** | 14/30  (46.67) | 13/18  (72.22) | 0.133 | 18/32  (56.25) | 9/16  (56.25) | 1.000 |
| **Death of sibling** | 2/30  (6.67) | 0/18  (0.00) | 0.521 | 1/32  (3.12) | 1/16  (6.25) | 1.000 |
| **Death of mother** | 0/30  (0.00) | 1/18  (5.56) | 0.375 | 0/32  (0.00) | 1/16  (6.25) | 0.333 |
| **Mother attended English school** | 5/30  (16.67) | 4/18  (22.22) | 0.711 | 6/32  (18.75) | 3/16  (18.75) | 1.000 |
| **Parents are monogamous** | 4/17  (23.53) | 4/8  (50.00) | 0.359 | 6/15  (40.00) | 2/10  (20.00) | 0.402 |
| **From core village** | 19/30  (63.33) | 9/18  (50.00) | 0.385 | 22/32  (68.75) | 6/16  (37.50) | 0.062 |
| **Only child** | 3/29  (10.34) | 0/16  (0.00) | 0.542 | 3/31  (9.68) | 0/14  (0.00) | 0.541 |
