# Supplementary material for: Factors Affecting Access to Healthcare: An Observational Study of Children under 5 Years of Age Presenting to a Rural Gambian Primary Healthcare Centre
Source: PLoS One. 2016 Jun 23;11(6):e0157790. doi: 10.1371/journal.pone.0157790 (PMC4919103; doi:10.1371/journal.pone.0157790)
Supplement: S5 Table — (DOCX) [file pone.0157790.s009.docx]

**S5 Table**

**Attendances with malaria- results of multivariate logistic regression for factors identified as significant in univariate regression analysis.**

| **Presentation type** | **Identified variable using univariate analysis** | **Unadjusted** | | **Adjusted for other variables significant in univariate analysis** | | **Adjusted for other variables significant in univariate analysis and seasonality** | | **Adjusted for other variables significant in univariate analysis and seasonality and year** | |
| --- | --- | --- | --- | --- | --- | --- | --- | --- | --- |
|  |  | **OR [95% CI]** | **p-value** | **OR [95% CI]** | **p-value** | **OR [95% CI]** | **p-value** | **OR [95% CI]** | **p-value** |
| **Delayed presentation** | Non identified in univariate analysis | N/A | N/A | N/A | N/A | N/A | N/A | N/A | N/A |
| **Severe illness** | Mother’s age | 1.131 [0.984, 1.300] | 0.084 | 0.998, [0.817, 1.220] | 0.985 | 1.036 [0.824, 1.302] | 0.763 | 1.052 [0.831, 1.331] | 0.673 |
|  | Number of maternal siblings | 1.480 [1.068, 20.049] | 0.018 | 3.094, [0.691, 13.858] | 0.140 | 2.205 [0.438, 11.105] | 0.338 | 2.426 [0.417, 14.113] | 0.324 |
|  | Birth order | 1.360 [1.026, 1.804] | 0.032 | 0.481, [0.131, 1.763] | 0.270 | 0.648 [0.164, 2.558] | 0.536 | 0.530 [0.116, 2.427] | 0.413 |
|  | From core village | 0.273 [0.077, 0.960] | 0.043 | 0.500, [0.112, 2.237] | 0.365 | 0.478 [0.096, 2.384] | 0.368 | 0.429 [0.078, 2.363] | 0.331 |
