# Supplementary material for: Factors Affecting Access to Healthcare: An Observational Study of Children under 5 Years of Age Presenting to a Rural Gambian Primary Healthcare Centre
Source: PLoS One. 2016 Jun 23;11(6):e0157790. doi: 10.1371/journal.pone.0157790 (PMC4919103; doi:10.1371/journal.pone.0157790)
Supplement: S6 Table — (DOCX) [file pone.0157790.s010.docx]

**S6 Table**

**Attendances with LRTI- results of univariate analysis of continuous independent variables.**

| **Continuous independent variables** | **n** | **Mean difference prompt vs. delayed [95% CI]** | **t-test**  **p-value** | **Mean difference non-severe vs. severe [95% CI]** | **t-test**  **p-value** |
| --- | --- | --- | --- | --- | --- |
| **Distance to clinic (km)** | 208 | -2.065 [-3.979, -0.151] | 0.035 | -1.705 [-3.787, 0.377] | 0.108 |
| **Child’s age (months)** | 208 | 3.081 [-1.259, 7.421] | 0.163 | 1.795 [-2.922, 6.511] | 0.454 |
| **Mother’s age (years)** | 204 | 0.086 [-1.968, 2.140] | 0.934 | -0.567 [-2.808, 1.675] | 0.619 |
