# Supplementary material for: Factors Affecting Access to Healthcare: An Observational Study of Children under 5 Years of Age Presenting to a Rural Gambian Primary Healthcare Centre
Source: PLoS One. 2016 Jun 23;11(6):e0157790. doi: 10.1371/journal.pone.0157790 (PMC4919103; doi:10.1371/journal.pone.0157790)
Supplement: S7 Table — (DOCX) [file pone.0157790.s011.docx]

**S7 Table**

**Attendances with LRTI- results of univariate analysis of ordered categorical independent variables.**

| **Order categorical variable** | **n** | **Delayed vs. non-delayed**  **Kruskal-Wallis one way analysis**  **Chi-squared with ties** | **p- value** | **Severe vs. non-severe**  **Kruskal-Wallis one way analysis Chi-squared with ties** | **p- value** |
| --- | --- | --- | --- | --- | --- |
| **Number of maternal siblings** | 204 | 0.208 with 1 d.f. | 0.649 | 1.094 with 1 d.f. | 0.296 |
| **Birth order** | 204 | 0.135 with 1 d.f. | 0.713 | 1.550 with 1 d.f. | 0.213 |
