# Supplementary material for: Factors Affecting Access to Healthcare: An Observational Study of Children under 5 Years of Age Presenting to a Rural Gambian Primary Healthcare Centre
Source: PLoS One. 2016 Jun 23;11(6):e0157790. doi: 10.1371/journal.pone.0157790 (PMC4919103; doi:10.1371/journal.pone.0157790)
Supplement: S8 Table — (DOCX) [file pone.0157790.s012.docx]

**S8 Table**

**Attendances with LRTI- results of univariate analysis of dichotomous independent variables.**

| **Dichotomous independent variables** | **Proportion prompt with variable (%)** | **Proportion delayed with variable (%)** | **Chi2 test**  **p-value** | **Proportion non-severe with variable (%)** | **Proportion severe with variable (%)** | **Chi2 test**  **p-value** |
| --- | --- | --- | --- | --- | --- | --- |
| **Severe illness** | 35/114  (30.70) | 28/94  (29.79) | 0.886 | N/A | N/A | N/A |
| **Delayed presentation** | N/A | N/A | N/A | 66/145  (45.52) | 28/63  (44.44) | 0.886 |
| **Male** | 63/114  (55.26) | 46/94  (48.94) | 0.363 | 73/145  (50.34) | 36/63  (57.14) | 0.367 |
| **Death of sibling** | 7/114  (6.14) | 11/94  (11.70) | 0.156 | 11/145  (7.59) | 7/63  (11.11) | 0.406 |
| **Death of mother** | 0/114  (0.00) | 0/94  (0.00) | N/A | 0/145  (0.00) | 0/63  (0.00) | N/A |
| **Mother attended English school** | 13/114  (11.40) | 17/93  (18.28) | 0.162 | 23/144  (15.97) | 7/63  (11.11) | 0.361 |
| **Parents are monogamous** | 14/67  (20.90) | 4/51  (7.84) | 0.051 | 14/83  (16.87) | 4/35  (11.43) | 0.453 |
| **From core village** | 69/114  (60.53) | 38/94  (40.43) | 0.004 | 82/145  (56.55) | 25/63  (39.68) | 0.025 |
| **Only child** | 12/112  (10.71) | 7/92  (7.61) | 0.448 | 15/144  (10.42) | 4/60  (6.67) | 0.401 |
