# Supplementary material for: Factors Affecting Access to Healthcare: An Observational Study of Children under 5 Years of Age Presenting to a Rural Gambian Primary Healthcare Centre
Source: PLoS One. 2016 Jun 23;11(6):e0157790. doi: 10.1371/journal.pone.0157790 (PMC4919103; doi:10.1371/journal.pone.0157790)
Supplement: S9 Table — (DOCX) [file pone.0157790.s013.docx]

**S9 Table**

**Attendances with LRTI- results of multivariate logistic regression for factors identified as significant in univariate regression analysis.**

| **Presentation type** | **Identified variable using univariate analysis** | **Unadjusted** | | **Adjusted for other variables significant in univariate analysis** | | **Adjusted for other variables significant in univariate analysis and seasonality** | | **Adjusted for other variables significant in univariate analysis and seasonality and year** | |
| --- | --- | --- | --- | --- | --- | --- | --- | --- | --- |
|  |  | **OR [95%CI]** | **p-value** | **OR [95% CI]** | **p-value** | **OR [95% CI]** | **p-value** | **OR [95% CI]** | **p-value** |
| **Delayed presentation** | ‘Core village’ | 0.443 [0.253, 0.773] | 0.004 | 1.122, [0.357, 3.529] | 0.844 | 1.173, [0.365, 3.769] | 0.789 | 1.352 [0.404, 4.520] | 0.625 |
|  | Distance to clinic | 1.043 [1.003, 1.086] | 0.036 | 1.058, [0.976, 1.147] | 0.173 | 1.064, [0.979, 1.156] | 0.142 | 1.063 [0.977, 1.158] | 0.158 |
|  | Parents are monogamous | 0.322 [0.099, 1.047] | 0.060 | 0.314, [0.094, 1.045] | 0.059 | 0.353, [0.103, 1.206] | 0.097 | 0.559 [0.152, 2.054] | 0.381 |
| **Severe illness** | ‘Core village’ | 0.505 [0.277, 0.923] | 0.026 | 0.505, [0.277,0.923] | 0.026 | 0.440, [0.235, 0.824] | 0.010 | 0.425 [0.225, 0.800] | 0.008 |
