# Supplementary material for: Factors Affecting Access to Healthcare: An Observational Study of Children under 5 Years of Age Presenting to a Rural Gambian Primary Healthcare Centre
Source: PLoS One. 2016 Jun 23;11(6):e0157790. doi: 10.1371/journal.pone.0157790 (PMC4919103; doi:10.1371/journal.pone.0157790)
Supplement: S10 Table — (DOCX) [file pone.0157790.s014.docx]

S10 Table

Attendances with diarrhoeal disease- results of univariate analysis of continuous independent variables

| **Continuous independent variables** | **n** | **Mean difference prompt vs. delayed [95% CI]** | **t-test**  **P-value** | **Mean difference non-severe vs. severe [95% CI]** | **t-test**  **P-value** |
| --- | --- | --- | --- | --- | --- |
| **Distance to clinic (km)** | 442 | -3.550 [-5.005, -2.096] | 0.000 | -1.829 [-4.968, 1.309] | 0.253 |
| **Child’s age (months)** | 442 | 1.674 [-0.652 4.001] | 0.158 | -2.394 [-7.301, 2.512] | 0.338 |
| **Mother’s age (years)** | 437 | 0.284 [-0.994, 1.562] | 0.663 | -0.299 [-2.977, 2.378] | 0.826 |
