# Supplementary material for: Factors Affecting Access to Healthcare: An Observational Study of Children under 5 Years of Age Presenting to a Rural Gambian Primary Healthcare Centre
Source: PLoS One. 2016 Jun 23;11(6):e0157790. doi: 10.1371/journal.pone.0157790 (PMC4919103; doi:10.1371/journal.pone.0157790)
Supplement: S11 Table — (DOCX) [file pone.0157790.s015.docx]

**S11 Table**

**Attendances with diarrhoeal disease- results of univariate analysis of ordered categorical independent variables**

| **Order categorical variable** | **n** | **Delayed vs. non-delayed**  **Kruskal-Wallis one way analysis**  **Chi-squared with ties** | **p- value** | **Severe vs. non-severe**  **Kruskal-Wallis one way analysis Chi-squared with ties** | **p- value** |
| --- | --- | --- | --- | --- | --- |
| **Number of maternal siblings** | 437 | 0.000 with 1 d.f. | 0.986 | 0.221 with 1 d.f. | 0.638 |
| **Birth order** | 436 | 0.019 with 1 d.f. | 0.889 | 0.112 with 1 d.f. | 0.738 |
