# Supplementary material for: Factors Affecting Access to Healthcare: An Observational Study of Children under 5 Years of Age Presenting to a Rural Gambian Primary Healthcare Centre
Source: PLoS One. 2016 Jun 23;11(6):e0157790. doi: 10.1371/journal.pone.0157790 (PMC4919103; doi:10.1371/journal.pone.0157790)
Supplement: S12 Table — (DOCX) [file pone.0157790.s016.docx]

**S12 Table**

**Attendances with diarrhoeal disease- results of univariate analysis of dichotomous independent variables**

| **Dichotomous independent variables** | **Proportion prompt with variable (%)** | **Proportion delayed with variable (%)** | **Chi2 test**  **P-value** | **Proportion non-severe with variable (%)** | **Proportion severe with variable (%)** | **Chi2 test**  **P-value** |
| --- | --- | --- | --- | --- | --- | --- |
| **Severe illness** | 20/250  (8.00) | 6/192  (3.12) | 0.031 | N/A | N/A | N/A |
| **Delayed presentation** | N/A | N/A | N/A | 186/416  (44.71) | 6/26  (23.08) | 0.031 |
| **Male** | 133/250  (53.20) | 102/192  (53.12) | 0.988 | 223/416  (53.61) | 12/26  (46.15) | 0.460 |
| **Death of sibling** | 17/250  (6.80) | 4/192  (2.08) | 0.021 | 17/416  (4.09) | 4/26  (15.38) | 0.009 |
| **Death of mother** | 2/250  (0.80) | 1/192  (0.52) | 0.723 | 3/416  (0.72) | 0/26  (0.00) | 0.664 |
| **Mother attended English school** | 46/250  (18.40) | 36/192  (18.75) | 0.925 | 76/416  (18.27) | 6/26  (23.08) | 0.541 |
| **Parents are monogamous** | 11/113  (9.73) | 6/94  (6.38) | 0.382 | 16/193  (8.29) | 1/14  (7.14) | 0.880 |
| **From core village** | 142/250  (56.80) | 66/192  (34.38) | 0.000 | 196/416  (47.12) | 12/26  (46.15) | 0.924 |
| **Only child** | 28/248  (11.29) | 29/189  (15.34) | 0.213 | 55/411  (13.38) | 2/26  (7.69) | 0.403 |
