# Supplementary material for: Factors Affecting Access to Healthcare: An Observational Study of Children under 5 Years of Age Presenting to a Rural Gambian Primary Healthcare Centre
Source: PLoS One. 2016 Jun 23;11(6):e0157790. doi: 10.1371/journal.pone.0157790 (PMC4919103; doi:10.1371/journal.pone.0157790)
Supplement: S13 Table — (DOCX) [file pone.0157790.s017.docx]

**S13 Table**

**Attendances with diarrhoeal disease- results of multivariate logistic regression for factors identified as significant in univariate regression analysis.**

| **Presentation type** | **Identified variable using univariate analysis** | **Unadjusted** | | **Adjusted for other variables significant in univariate analysis** | | **Adjusted for other variables significant in univariate analysis and seasonality** | | **Adjusted for other variables significant in univariate analysis and seasonality and year** | |
| --- | --- | --- | --- | --- | --- | --- | --- | --- | --- |
|  |  | **OR [95%CI]** | **p-value** | **OR [95% CI]** | **p-value** | **OR [95% CI]** | **p-value** | **OR [95% CI]** | **p-value** |
| **Delayed presentation** | Distance to clinic | 1.060, [1.034, 1.087] | 0.000 | 1.039, [1.002,1.077] | 0.036 | 1.034, [0.997, 1.072] | 0.075 | 1.034 [0.997, 1.073] | 0.070 |
|  | Severe illness | 0.371, [0.146, 0.943] | 0.037 | 0.353, [0.133,0.932] | 0.036 | 0.330, [0.122, 0.890] | 0.028 | 0.314 [0.116, 0.851] | 0.023 |
|  | ‘Core village’ | 0.398, [0.270, 0.588] | 0.000 | 0.603, [0.346,1.052] | 0.075 | 0.563, [0.318, 0.994] | 0.047 | 0.559 [0.316, 0.988] | 0.045 |
|  | Dead sibling | 0.292, [0.096, 0.881] | 0.029 | 0.317, [0.101,0.992] | 0.048 | 0.294, [0.091, 0.948] | 0.040 | 0.297 [0.092, 0.960] | 0.042 |
| **Severe illness** | Delayed presentation | 0.371, [0.146, 0.943] | 0.037 | 0.406, [0.158,1.042] | 0.061 | 0.397, [0.152, 1.034] | 0.059 | 0.378 [0.144, 0.992] | 0.048 |
|  | Dead sibling | 4.267 ,[1.324, 13.759] | 0.015 | 3.557, [1.086,11.652] | 0.036 | 3.706, [1.112, 12.355] | 0.033 | 3.909 [1.167, 13.096] | 0.027 |
