# Supplementary material for: Factors Affecting Access to Healthcare: An Observational Study of Children under 5 Years of Age Presenting to a Rural Gambian Primary Healthcare Centre
Source: PLoS One. 2016 Jun 23;11(6):e0157790. doi: 10.1371/journal.pone.0157790 (PMC4919103; doi:10.1371/journal.pone.0157790)
Supplement: S15 Table — (DOCX) [file pone.0157790.s019.docx]

**S19 Table**

**List of ICD-10 codes in KEMReS during study period**

| **ICD 10 Code** | **Short diagnostic label** |
| --- | --- |
| A00 | Cholera |
| A01.0 | Typhoid fever |
| A02.0 | Salmonella enteritis |
| A03 | Shigellosis |
| A04 | Other bacterial intestinal infections |
| A04.9 | Bacterial intestinal infection |
| A06 | Amoebiasis |
| A07.1 | Giardia |
| A07.2 | Cryptosporidiosis |
| A08 | Viral Gastroenteritis |
| A15 | Tuberculosis |
| A17.0 | Tuberculosis of bones and joints |
| A30 | Leprosy |
| A36 | Diphtheria |
| A41.9 | Septicaemia |
| A53.9 | Syphilis |
| A54 | Neisserra gonorrhea |
| A57 | Chancroid |
| A59 | Trichomoniasis |
| A59.0 | Trichomoniasis, urogenital |
| A64 | Sexually transmitted disease |
| A80 | Acute poliomyelitis |
| A82 | Rabies |
| A85 | Viral encephalitis |
| A87 | Viral Meningitis |
| B00 | Herpes infections |
| B00.1 | Cold sore |
| B00.9 | Herpes viral infection, unspecified |
| B01 | Varicella (chickenpox) |
| B05 | Measles |
| B08.1 | Molluscum contagiosum |
| B16 | Acute Hepatitis B |
| B18.1 | Chronic Hepatitis B |
| B19 | Viral hepatitis |
| B20 | Symptomatic HIV infection |
| B26 | Mumps |
| B30.9 | Viral conjunctivitis |
| B34.9 | Viral infection of unspecified site |
| B35.9 | Ring Worm |
| B37 | Oral candidiasis |
| B37.3 | Candidiasis of vulva and vagina |
| B37.9 | Candidiasis |
| B50 | Plasmodium falciparum malaria |
| B50.0 | Cerebral malaria |
| B51 | Plasmodium vivax malaria |
| B55 | Leishmaniasis |
| B56 | Trypanosomiasis |
| B65 | Schistosomiasis, unspecified |
| B76 | Hookworm disease |
| B79 | Trichuriasis |
| B82.0 | Intestinal helminthiasis |
| B86 | Scabies |
| B91 | Sequelae of Poliomyelitis |
| C22 | Liver tumour |
| C34 | Lung tumour |
| C62.9 | Malignant neoplasm of Testis |
| C81 | Hodgkin's disease |
| C85.9 | Non-Hodgkin's lymphoma |
| C95 | Leukaemia |
| D29.2 | Benign neoplasm of Testis |
| D53.9 | Anaemia |
| D57 | Sickle cell disorders |
| D59.2 | Haemolytic-uraemic syndrome |
| E03.9 | Hypothyroidism |
| E04.9 | Nontoxic goitre |
| E05 | Hyperthyroidism |
| E10 | Insulin-dependent diabetes mellitus |
| E10.1 | Diabetic ketoacidosis |
| E10.3 | Diabetic retinopathy |
| E10.4 | Diabetic neuropathy |
| E11 | Non-insulin-dependent diabetes mellitus |
| E16.2 | Hypoglycaemia |
| E20 | Hypoparathyroidism |
| E21 | Hyperparathyroidism |
| E23.7 | Pituitary gland disorders |
| E24 | Cushing's syndrome |
| E26 | Hyperaldosteronism |
| E27.1 | Addisons's disease |
| E29.9 | Testicular dysfuntion |
| E40 | Kwashiorkor |
| E41 | Marasmus |
| E42 | Marasmic kwashiorkor |
| E43 | Severe protein-energy malnutrition |
| E45 | Stunting |
| E54 | Vitamin C deficiency |
| E55.0 | Rickets |
| E66 | Obesity |
| E83.0 | Wilson's disease |
| E86 | Volume depletion/Dehydration |
| F03 | Dementia |
| F05 | Delirium |
| F20 | Schizophrenia |
| F29 | Psychosis |
| F32.9 | Depression |
| F41.9 | Anxiety disorder |
| F51.9 | Insomnia |
| F52 | Sexual dysfunction |
| F98.0 | Nonorganic enuresis |
| G00 | Bacterial Meningitis |
| G03.9 | Meningitis |
| G04.0 | Tropical spastic paraplegia |
| G20 | Parkinson's disease |
| G40 | Epilepsy |
| G41 | Status Epilepticus |
| G43.9 | Migraine |
| G44.2 | Tension-type headache |
| G45 | Transient Ischaemic Attack |
| G51.0 | Bell's palsy |
| G61 | Guillain-Barre syndrome |
| G70 | Myasthenia gravis |
| G71.0 | Muscular dystrophy |
| G80 | Cerebral palsy |
| G93.2 | Benign intracranial hypertension |
| H01.9 | Stye |
| H02.0 | Entropion and trichiasis of eyelid |
| H02.4 | Ptosis of eyelid |
| H05.0 | Orbital or periobital cellulitis |
| H10.1 | Allergic Conjunctivitis |
| H10.4 | Chronic conjunctivitis |
| H10.9 | Conjunctivitis |
| H11.0 | Pterygium |
| H17.9 | Corneal scar and opacity |
| H20 | Iridocyclitis |
| H21.0 | Hyphaema |
| H25 | Senile cataract |
| H26.9 | Cataract |
| H53.1 | Subjective visual disturbances |
| H60 | Otitis externa |
| H61.2 | Wax in ear |
| H65 | Nonsuppurative Otitis Media |
| H66.0 | Suppurative otitis media |
| H66.3 | Chronic suppurative otitis media |
| H83.0 | Labyrinthitis |
| I00 | Acute Rheumatic Fever |
| I09.0 | Rheumatic heart disease |
| I10 | Hypertension |
| I20 | Angina pectoris |
| I21 | Myocardial infarction |
| I25 | Ischaemic heart disease |
| I26 | Pulmonary embolism |
| I27.9 | Pulmonary heart disease |
| I30 | Pericarditis |
| I33 | Infective endocarditis |
| I38 | Endocarditis |
| I42 | Cardiomyopathy |
| I48 | Atrial fibrillation and flutter |
| I49.9 | Cardiac arrhythmias |
| I50 | Heart Failure |
| I60 | Subarachnoid haemorrhage |
| I62.0 | Subdural haemorrhage |
| I64 | Stroke |
| I67.9 | Cerebrovascular disease |
| I83 | Varicose Veins |
| I84 | Haemorrhoids |
| I88.0 | Mesenteric lymphadenitis |
| I95.9 | Hypotension |
| J00 | Common Cold |
| J02.9 | Pharyngitis |
| J03 | Tonsillitis |
| J03.9 | Tonsillitis |
| J05.0 | Croup |
| J05.1 | Epiglottitis |
| J18 | Pneumonia, organism unspecified |
| J20 | Acute bronchitis |
| J21 | Acute bronchiolitis |
| J32 | Chronic sinusitis |
| J36 | Peritonsillar abscess |
| J42 | Chronic bronchitis |
| J44.9 | COPD |
| J45 | Asthma |
| J46 | Status asthmaticus |
| J47 | Bronchiectasis |
| J90 | Pleural effusion |
| J93 | Pneumothorax |
| J96 | Respiratory Failure |
| K02 | Dental caries |
| K04.7 | Dental abscess |
| K05 | Gingivitis |
| K05.0 | Acute gingivitis |
| K08.8 | Touthache |
| K12 | Stomatitis |
| K12.0 | Aphthous Ulcers |
| K13.0 | Cheilitis |
| K13.2 | Leukoplakia |
| K14.0 | Glossitis |
| K21 | Gastro-oesophageal reflux disease |
| K22.6 | Mallory-Weiss syndrome |
| K27 | Peptic Ulcer Disease |
| K29.7 | Gastritis |
| K30 | Dyspepsia |
| K40 | Inguinal hernia |
| K42 | Umbilical hernia |
| K52.9 | Non infective diarrhoea |
| K56.1 | Intussusception |
| K58 | Irritable bowel syndrome |
| K59.0 | Constipation |
| K62.3 | Rectal Prolapse |
| K72 | Liver failure |
| K74 | Liver Cirrhosis |
| K74.3 | Primary biliary cirrhosis |
| K80.8 | Cholelithiasis |
| K81.9 | Cholecystitis |
| L01 | Impetigo |
| L02.9 | Cutaneous abscess, furuncle and carbuncle |
| L03 | Cellulitis |
| L03.0 | Paronychia |
| L04.9 | Lymphadenitis |
| L08.9 | Local infection of skin & subcutaneous tissue |
| L20.8 | Eczema |
| L21 | Seborrhoeic dermatitis |
| L25 | Unspecified contact dermatitis |
| L28.2 | Prurigo |
| L40.9 | Psoriasis |
| L50.0 | Allergic urticaria |
| L50.9 | Urticaria |
| L63.9 | Alopecia areata |
| L85.3 | Dry skin dermatitis |
| M06.9 | Rheumatoid arthritis |
| M10 | Gout |
| M13.9 | Arthritis |
| M43.6 | Torticollis |
| M51.9 | Intervertebral disc disorder |
| M54.5 | Back pain |
| M54.9 | Backache |
| N00 | Acute nephritic syndrome |
| N04 | Nephrotic syndrome |
| N10 | Pyelonephritis |
| N17 | Acute Renal Failure |
| N17.0 | Acute renal failure with tubular necrosis |
| N18 | Chronic renal failure |
| N20 | Renal Stones |
| N20.9 | Urinary calculus |
| N34.1 | Urethritis |
| N39.0 | Urinary tract infection |
| N40 | Hyperplasia of prostate |
| N46 | Male infertility |
| N47 | Paraphimosis |
| N48.4 | Impotence |
| N73.9 | Pelvic inflammatory disease |
| N81.1 | Cystocele |
| N91.2 | Amenorrhoea |
| N92.6 | Irregular menstruation |
| N94.3 | Premenstrual tension syndrome |
| N94.6 | Dysmenorrhoea |
| N95.9 | Meno-and perimenopausal disorder |
| N97.9 | Female infertility |
| O06 | Unspecified abortion |
| O21.9 | Hyperemesis gravidarum |
| O91.2 | Non-purulent mastitis |
| O92.0 | Retracted nipple associated with childbirth |
| P12.0 | Cephalhaematoma due to birth injury |
| P21 | Birth asphyxia |
| P36 | Bacterial sepsis of newborn |
| P39.1 | Neonatal conjunctivitis |
| P39.4 | Neonatal skin infection |
| P58.2 | Neonatal jaundice due to infection |
| P83.5 | Congenital hydrocele |
| Q05 | Spina bifida |
| Q25 | Congenital heart disease |
| Q31.5 | Congenital laryngomalacia |
| Q44.2 | Congenital biliary atresia |
| Q77.4 | Achondrolasia |
| Q82.2 | Mastocytosis |
| Q82.8 | Skin tags |
| Q90 | Down syndrome |
| R01.0 | Benign cardia murmur |
| R04.0 | Epistaxis |
| R04.2 | Haemoptysis |
| R06.6 | Hiccough |
| R10.1 | Epigastric Pain |
| R10.4 | Abdominal Pain |
| R12 | Heart burn |
| R21 | Unspecified Rash |
| R33 | Retention of urine |
| R50 | Fever of unkown origin |
| R50.9 | Persistent Hyperpyrexia |
| R51 | Headache |
| R52.9 | Unspecified pain |
| R53 | Malaise and fatigue |
| R54 | Old Age |
| R56.0 | Febrile convulsion |
| R57.9 | Shock |
| R59.1 | Lymphadenopathy |
| R62 | Lack of expected normal physiological development |
| R69 | Unknown Diagnosis |
| R90.0 | Intracranial space-occupying lesion |
| S09.9 | Head injury |
| S69.9 | Injury to hand or wrist |
| S73.0 | Dislocation of hip |
| T14.0 | Superficial injury |
| T14.9 | Injury |
| T16 | Foreign body in ear |
| T17 | Foreign body in respiratory tract |
| T20 | Burn of head and neck |
| T21 | Burn of trunk |
| T22.0 | Burn of shoulder and upper limb |
| T23 | Burn of wrist and hand |
| T24 | Burn of hip and lower limb |
| T25 | Burn of ankle and foot |
| T50.9 | Poisoning |
| T63.2 | Scorpion bite |
| T78.4 | Allergy |
| T79.3 | Post injury infected wound |
| V99 | Road traffic accident |
| W19 | Fall |
| W44 | Foreign body in eye |
| W54 | Dog bite |
| X49 | Accidental poisoning |
| Z00.1 | Routine child health examination |
| Z09 | Follow up examination |
| Z21 | Asymptomatic HIV infection |
| Z48.9 | Follow up Dressing |
